# Highly Potent Fluorogenic Ligands for Triplex DNA: 5‐Substituted 2‐(Naphthalen‐2‐yl)‐4*H*‐Chromen‐4‐Ones

**DOI:** 10.1002/chem.202503318

**Published:** 2026-01-12

**Authors:** Nghia Tran, Landy Gu, Krege M. Christison, Kalee Thao, Liang Xue

**Affiliations:** ^1^ Department of Chemistry University of the Pacific Stockton California USA

**Keywords:** antigene enhancers, flavonoids, fluorogenic probes, ligand‐mediated triplex formation, triplex‐specific ligands

## Abstract

We previously reported two series of 5‐substituted flavonoids that selectively bind to triplex DNA while interacting minimally with duplex DNA. Our studies suggest that flavonoids are a promising scaffold for designing triplex ligands, owing to their mild synthesis conditions and ease of structural modification. In the present work, we introduce a novel class of highly potent triplex‐specific ligands [5‐substituted 2‐(naphthalen‐2‐yl)‐4*H*‐chromen‐4‐ones], developed by expanding the aromatic surface of the flavone moiety. UV thermal denaturation studies indicate these compounds strongly stabilize triplex DNAs, enabling their direct dissociation into random coils without transitioning through a duplex intermediate. Circular dichroism (CD) analysis reveals that these ligands induce much greater conformational changes in triplex DNA compared to duplex DNA. Fluorescent intercalator (FID) displacement assays cross‐verify the importance of pendant chains for triplex binding. Viscosity measurements suggest that these compounds bind to triplex DNA via intercalation. Notably, a representative compound (**3b**) emits strong blue fluorescence upon binding to triplex DNA, allowing direct visualization under UV illumination and providing a convenient method for probing triplex DNA. Electrophoretic mobility shift assay confirms that **3b** promotes triplex formation at submicromolar concentrations. Additionally, a plasmid‐based assay developed in our lab demonstrates that **3b** inhibits *DraI* endonuclease activity at low micromolar concentrations.

## Graphical abstract

5‐Substituted 2‐(naphthalen‐2‐yl)‐4*H*‐chromen‐4‐ones are reported as a novel class of highly potent and selective triplex DNA ligands. These ligands induce triplex formation at submicromolar concentrations and inhibit enzymatic activity via ligand‐mediated triplex formation. Binding to triplex DNA triggers an intense blue fluorescence emission in representative compound **3b**, whereas no significant fluorescence emission occurs with duplex DNA.

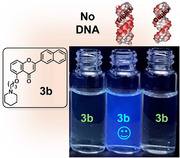

## 1 Introduction

For many years, researchers have focused on targeting duplex DNA in a sequence‐specific approach to regulate transcription or replication, a key area in drug development [1]. DNA sequences of 15–16 nucleotides or longer are statistically unique within the human genome, making them promising targets for achieving therapeutic selectivity and specificity [2, 3, 4, 5]. Triplex‐forming oligonucleotides (TFOs) are well‐suited for this purpose, as they can bind to duplex DNA with high specificity (also known as the antigene strategy) based on two base‐triplet motifs: T•A:T or C^+^•G:C for the pyrimidine motif and A•A:T or G•G:C for the purine motif [6]. However, triplex DNAs are less stable than their duplex counterparts and form at rates orders of magnitude slower, limiting their use as antigene agents [7, 8]. To address these challenges while leveraging TFO specificity for targeting duplex DNA, researchers have explored several approaches, including nucleobase modification, DNA backbone alterations, and the use of triplex‐specific ligands (small molecules). Professor Frank Seela is a notable pioneer in developing deaza‐type nucleobases to enhance triplex stability [9, 10, 11]. Other nucleobase modifications for promoting the formation of diverse triplex DNA structures have also been explored over the past two decades [12, 13, 14]. Alterations to the DNA backbone, for instance, converting phosphate to methylphosphonate or phosphorothioate, have been shown to influence triplex formation [15]. Ideal triplex‐specific ligands, also known as antigene enhancers, should bind preferentially to triplex DNA with minimal impact on duplex DNA. Over the past two decades, several classes of such ligands have been developed, including berenil derivatives [16, 17], coralynes [18], naphthylquinolines [19, 20], aminoglycosides [21, 22], and 5‐substituted flavonoid derivatives [23, 24]. However, reports on new triplex ligand scaffolds have dwindled in the last decade, with most advancements concentrated earlier. Discovering new structural motifs for triplex‐specific ligands could spark significant progress in the antigene strategy and related therapeutic applications.

**FIGURE 1:**
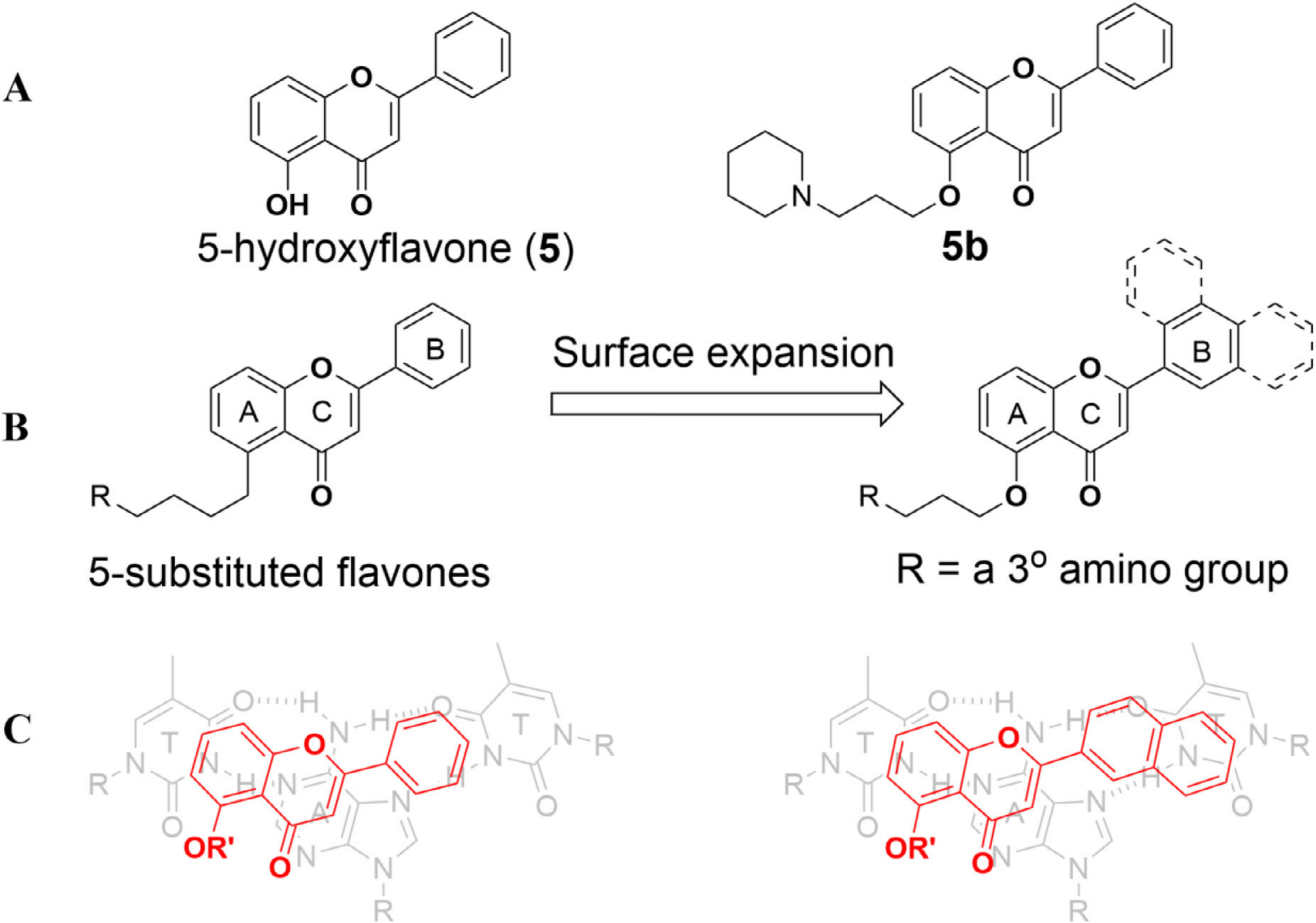
A) Structures of 5‐hydroxyflavone (**5**) and a representative 5‐substituted flavone derivative (**5b**). B) Demonstration of aromatic surface expansion for 5‐substituted flavones. Two plausible rings for expansion are shown as dotted lines, respectively. Detailed compound structures are shown in Scheme 1. C) Illustration of stacking a T•A:T triplet (grey) with 5‐substituted flavone (red, left) and 5‐substituted 2‐(naphthalen‐2‐yl)‐4*H*‐chromen‐4‐one (red, right). R′ = an amino‐containing side chain. Note: these images are intended to show stacking efficiency and do not represent the actual binding orientation.

**SCHEME 1:**
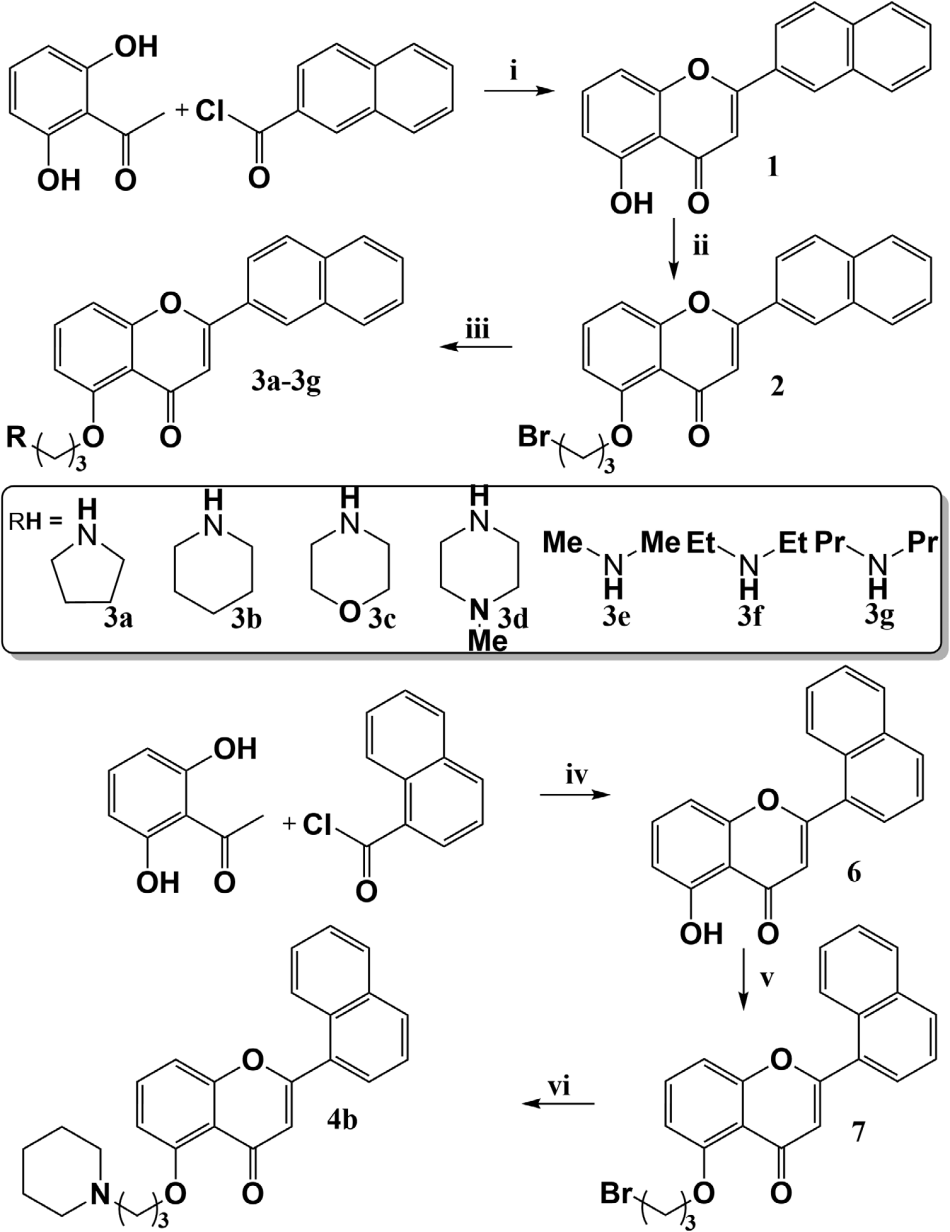
Synthesis of compounds **1, 2**, **3a‐3g**, **6**, **7**, and **4b**. i) K_2_CO_3_, acetone, reflux, 5 h, 23.0%; ii) Br(CH_2_)_3_Br, DMF, K_2_CO_3_, r.t., 4 d, 44.2%; iii) DMF, K_2_CO_3_, a 2° amine, r.t., 20–48 h, 56.1‐96.0%; iv) K_2_CO_3_, acetone, reflux, 3 h, 22.7%;v) Br(CH_2_)_3_Br, DMF, K_2_CO_3_, r.t., 4 d, 67.9%; vi) DMF, K_2_CO_3_, piperidine, r.t., 2 d, 87.7%;.

Our laboratory recently reported two classes of highly effective 5‐substituted flavonoid‐based ligands [23, 24] (a representative compound **5b** is shown in Figure 1A) that specifically bind to triplex DNA, with binding strengths equal to or better than those of renowned triplex ligands such as neomycin. The new scaffold's key advantage lies in its ease of modification under mild synthesis conditions, thereby enabling us to investigate critical factors in triplex DNA binding, such as side chains and end groups. We discovered that amino‐containing side chains at the 5‐position and an appropriate linker length significantly enhance triplex binding affinity and specificity [24]. In this study, we further modified the flavonoid scaffold and explored the factors that could lead to more potent triplex‐specific ligands. A consensus in the field is that the selectivity of triplex over duplex DNA can be largely affected by the stacking surface [25]. Herein, we modified the structures of previously reported 5‐substituted flavones by replacing the ring B (phenyl) with a naphthalenyl group to yield a series of 5‐substituted 2‐(naphthalenyl)‐4*H*‐chromen‐4‐ones (Figure 1B; detailed structures, **3a**‐**3g** and **4b**, are shown in Scheme 1). It should be noted that the tertiary amino groups in the side chain at the 5‐position of the flavone scaffold are denoted by the letters (a‐g) in compound symbols, as follows: pyrrolidine (a), piperidine (b), morpholine (c), *N*‐methylpiperazine (d), dimethylamine (e), diethylamine (f), and dipropylamine (g). Ligands with a larger aromatic surface, in general, stack better and more selectively to base triplets (in triplex) than base pairs (in duplex), as shown in Figure 1C. The binding of these newly synthesized molecules to triplex DNA was determined using various biophysical methods, including UV thermal denaturation, fluorescence emission, circular dichroism (CD), fluorescent intercalator displacement (FID), and a gel mobility shift assay. The inhibition of enzymatic activities via ligand‐mediated triplex formation was examined using a plasmid‐based assay established in our lab. The work presented here is a continuous effort from our laboratory to explore novel scaffolds of triplex‐specific ligands based on flavonoids.

## 2 Results and Discussion

### 2.1 Preparation of 5‐Substituted 2‐(Naphthalen‐2‐yl)‐4*H*‐Chromen‐4‐Ones

The synthesis of 5‐substituted 2‐(naphthalen‐2‐yl)‐4*H*‐chromen‐4‐ones was accomplished with a three‐step reaction scheme (Scheme 1). Coupling of 2′,6′‐dihydroxyacetophenone with 2‐naphthoyl chloride or 1‐naphthoyl chloride in the presence of K_2_CO_3_ yielded 5‐hydroxy‐2‐(naphthalen‐2‐yl)‐4*H*‐chromen‐4‐one (**1**) and 5‐hydroxy‐2‐(naphthalen‐1‐yl)‐4*H*‐chromen‐4‐one (**6**), respectively. Reaction of **1** with 1,3‐dibromopropane via a biomolecular nucleophilic substitution mechanism gave compound **2**. We chose a propylene linker in this study because it was previously shown to be optimal for studying 5‐substituted flavones in triplex binding. Replacement of the bromo group in **2** with a secondary amine, including pyrrolidine, piperidine, morpholine, *N*‐methylpiperazine, dimethylamine, diethylamine, and dipropylamine, yielded compounds **3a**‐**3g** (Scheme 1), respectively. Previous studies also suggest that the amino‐containing end groups of the side chains are important for triplex stabilization by 5‐substituted flavones. Removal of the nitrogen or replacement of the amino group with a hydroxyl group results in a drastically reduced triplex‐stabilization effect [24]. Following a similar synthetic scheme, reaction of **6** with 1,3‐dibromopropane under basic conditions yielded compound **7**. Replacement of the bromo group in **7** with piperidine yielded compound **4b**. Preparation of **4b** aims to determine the effect of surface curvature on ligand stacking with base triplets, a subject that has rarely been studied.

### 2.2 Thermal Stabilization of Triplex DNAs by 5‐Substituted 2‐(Naphthalen‐2‐yl)‐4*H*‐Chromen‐4‐Ones

Upon completing the synthesis, we sought to investigate their binding to triplex DNAs using UV thermal denaturation. Two types of triplex DNAs were used in this study, including a polynucleotide DNA triplex [poly(dA)•2poly(dT)] and an intramolecular DNA triplex (**DNA1**, full sequence shown in Figure 3D). Poly(dA)•2poly(dT) is a model triplex used for thermal denaturation, as it gives a well‐defined triplex dissociation curve. The use of **DNA1** allows us to verify that the observed stabilization effect also applies to short (more native‐like) DNA triplexes. In a typical experiment, UV spectra of a DNA triplex solution in the presence and absence of a ligand of interest (e.g., **1**, **3a**‐**3g**, **6**, **5b**, and **4b**) are monitored continuously at 260, 280, and 284 nm while being heated from 20 to 95°C [26]. The dissociation of triplex DNA involves two hyperchromic transitions. The transition at lower temperatures represents the dissociation of the triplex DNA into a duplex DNA and a single strand (TFO). The transition at higher temperatures represents the dissociation of the remaining duplex DNA into random coils. These transitions are designated as the melting temperature (T_m_) of DNA, the temperature at which 50% of the DNA in a sample has denatured. The symbols of T_m3→2_ and T_m2→1_ are defined as the melting temperature of triplex and duplex DNA, respectively. The ΔT_m_ is defined as the difference between the T_m_ values in the presence and absence of a ligand of interest. Spectra obtained at 260 nm are typically used for data processing because they provide the best signal‐to‐noise ratio. Spectra obtained at 280 and 284 nm are used to double‐check the dissociation of triplex and duplex (typically, 280 nm for triplex and 284 nm for duplex), as the melting profiles of triplex and duplex DNA at these two wavelengths are distinct.

**FIGURE 2:**
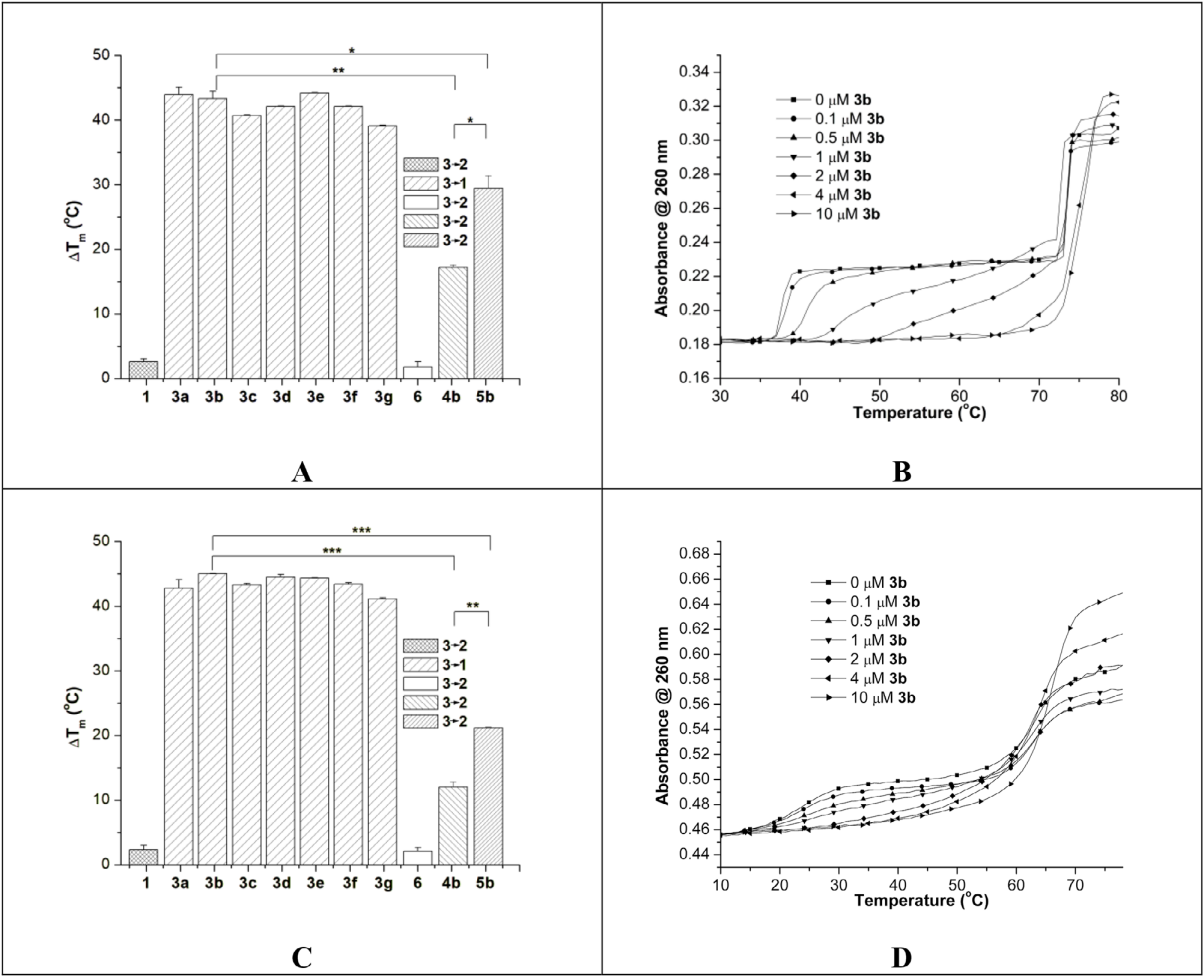
Changes in UV melting temperatures (ΔT_m3→1_ or ΔT_m3→2_) of poly(dA)•2poly(dT) (A) and **DNA3** (B) at 260 nm with 10 µM of a ligand (**1**, **3a‐3g**, **4b**, **5b**, and **6**). [DNA] = 15 µM/base triplet. The symbol 3→2 refers to the dissociation of triplex DNA into duplex DNA and 3→1 refers to the dissociation of triplex DNA into random coils. C) Melting profiles of poly(dA)•2poly(dT) in the presence of 0−10 µM **3b**. D) Melting profiles of **DNA1** in the presence of 0−10 µM **3b**. Buffer: 10 mM sodium cacodylate (pH 7.0), 150 mM KCl for poly(dA)•2poly(dT), and 100 mM NaCl for **DNA1**. The error bars represent the standard deviation of two experiments (n = 2). Statistical analysis was conducted using the two‐tailed unpaired t‐test. *P < 0.05 (significant); **P < 0.01 (very significant); ***P <0.001 (very significant).

The ΔT_m_ values of poly(dA)•2poly(dT) (15 µM/base triplet) in the presence of a ligand (**1**, **3a**‐**3g**, **6**, **5b**, and **4b**) at 10 µM are shown as a bar graph in Figure 2A. In the presence of the parent compound **1**, the ΔT_m3→2_ value is (2.6 ± 0.5) °C, suggesting that without the amino‐containing side chain at the 5‐position, the effect of compound **1** on triplex DNA is minimal. Under the same conditions, the stabilization of the duplex DNA, poly(dA)•poly(dT), was not observed. In the presence of any 5‐substituted 2‐(naphthalen‐2‐yl)‐4*H*‐chromen‐4‐ones (**3a**‐**3g**), the increments of the ΔT_m_ values are more than 39°C (Figure 2A). It is worth noting that we observed a single‐phase transition rather than the typical biphasic transition during triplex dissociation, suggesting direct dissociation of triplex DNA into random coils without passing through the triplex‐to‐duplex stage. The symbol of T_m3→1_ is designated for the melting temperature of this transition. Molecules inducing the direct dissociation of triplex DNA into random coils are considered extremely potent triplex‐specific ligands, which hold three strands so tightly that they cannot dissociate via a duplex intermediate. Hélène and his coworkers reported a rationally designed triplex‐specific ligand, BQQ, that promotes the dissociation of triplex DNA with a single‐phase transition, similar to our observations mentioned here [25]. It is worth noting that BQQ is considered one of the most potent triplex‐specific ligands developed so far. Wilson and his coworkers reported a similar observation on a naphthylquinoline derivative at the high ligand/DNA ratios [20].

To confirm that the observed transition indeed represents the dissociation of triplex DNA into random coils, we conducted a concentration‐dependent study on thermal denaturation of poly(dA)•2poly(dT) in the presence of **3b** with concentrations ranging from 0 to 10 µM. The melting profiles of poly(dA)•2poly(dT) are shown in Figure 2B. At lower concentrations of **3b**, the dissociation of poly(dA)•2poly(dT) is a clear biphasic transition. In the presence of 0.1 µM **3b**, the ΔT_m3→2_ value almost remained unchanged, and in the presence of 0.5 µM **3b**, the ΔT_m3→2_ value increased by 3°C. The 3→2 transitions continuously shifted to higher temperatures when the concentration of **3b** was 1 and 2 µM. At these two concentrations, the broadening of the 3→2 transitions was observed. In the presence of 4 µM of **3b**, the 3→2 transition almost merged with the 2→1 transition, and when the concentration of **3b** reached 10 µM, only the 3→1 transition was observed. The gradual changes in the melting profiles presented here unambiguously confirm that the observed single transition indeed represents the dissociation of triplex into random coils.

Even though all amino side chains confer excellent potency for triplex stabilization, we observed a slight difference in effect upon close examination (Figure 2A). The rank order of the side chain effect is **3a** ≈ **3b** ≈ **3e** > **3d** ≈ **3f** > **3**c > **3** **g**. For the compounds with a cyclic amino end group, the ones with pyrrolidine (**3a**), piperidine (**3b**), and methylpiperazine (**3e**) showed higher ΔT_m3→1_ values (> 42.1°C) than that with morpholine (**3c**) (40.7°C). For the compounds with a dialkylamino end group, the ones with dimethylamine (**3e**) and diethylamine (**3f**) showed higher ΔT_m3→1_ values (> 42.1°C) than the one with dipropylamine (**3** **g**) (39.1°C). Increasing the size of the dialkylamino group tends to reduce the ligand's triplex stabilization effect due to the steric hindrance of a larger end group in the DNA groove. The melting profile of poly(dA)•2poly(dT) in the presence of compound **5b** under the same conditions showed a biphasic transition with a ΔT_m3→2_ value of (29.4 ± 0.2) °C. Hence, compound **5b,** containing a flavone moiety, could not stabilize triplex DNA as significantly as compounds **3a**‐**3g** with an extended aromatic surface. This conclusion supports our hypothesis that expanding the aromatic surface of the flavonoid scaffold can be used for designing more potent triplex‐specific ligands.

The facile synthesis of the molecules above enables us to explore the effect of aromatic surface curvature on triplex stabilization, a subject that has not been extensively studied. Compound **6** (the parent compound of **4b** without a side chain) showed a minimal triplex stabilization effect with a ΔT_m_ value of (1.8 ± 0.8) °C for poly(dA)•2poly(dT) (Figure 2A), which aligns with our expectations. The melting profile of poly(dA)•2poly(dT) in the presence of **4b** is biphasic, with a ΔT_m3→2_ value of (17.2 ± 0.4) °C and a ΔT_m2→1_ value of (0.3 ± 0.2) °C. Under the same experimental conditions, compound **4b** stabilizes poly(dA)•2poly(dT) 26.1°C and 12.2°C less than compounds **3b** and **5b**, respectively. Interestingly, compound **4b** has the same surface area (size) as compound **3b**, but the surface curvatures are different. These results suggest that the aromatic surface area (size) is not the sole important factor when designing triplex‐specific ligands. The curvature (or orientation) of the aromatic surface also plays a crucial role in triplex stabilization. A correctly oriented aromatic surface (compound **3b**) could enhance the effective stacking surface between the ligand and base triplets. An ill‐oriented aromatic surface (compound **4b**) brings a detrimental effect on the triplex stabilization of ligands. In some cases, these ligands (e.g., **4b**) stabilize triplex DNA even less efficiently than ligands with smaller aromatic surface areas (e.g., **5b**).

**FIGURE 3:**
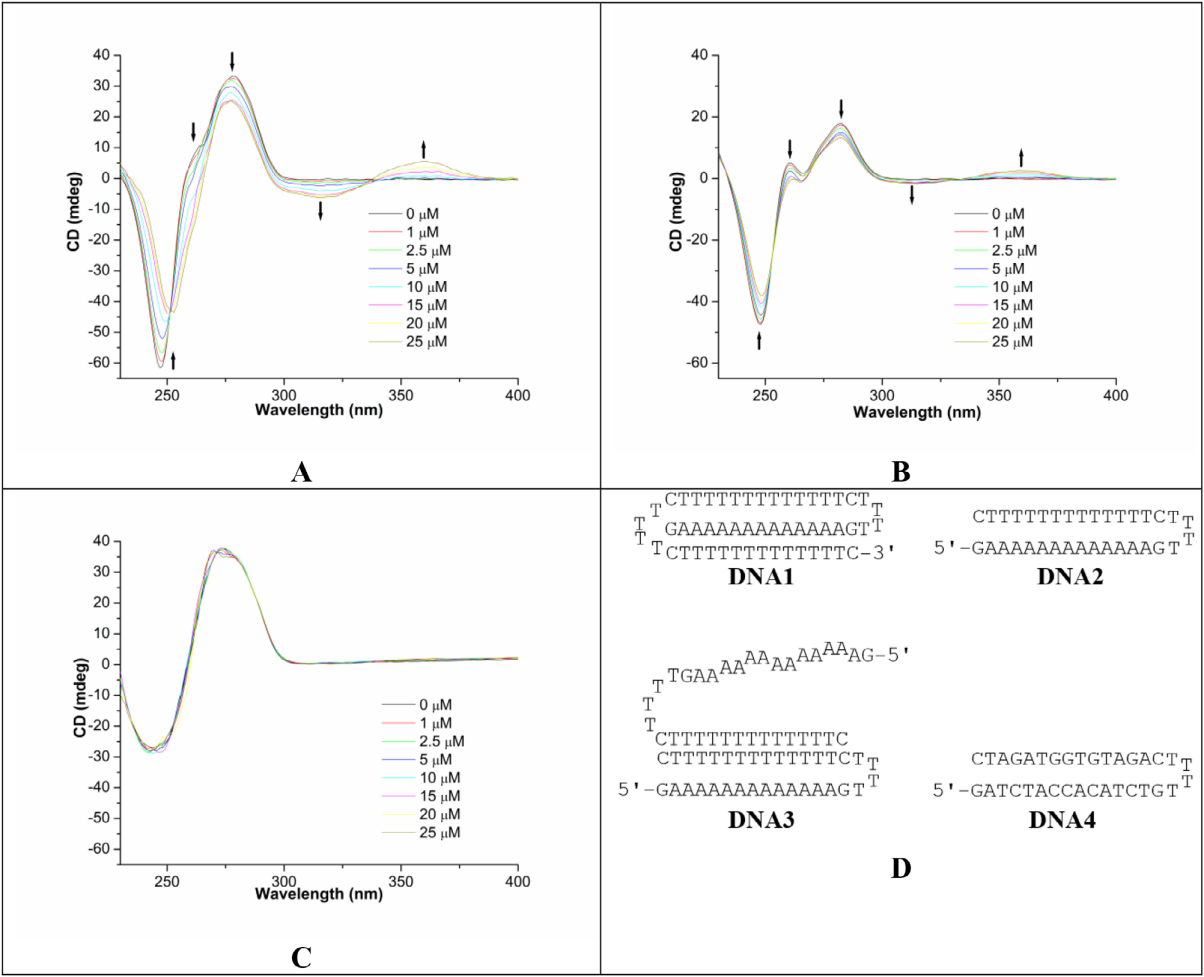
A) CD spectra of **DNA1** recorded when titrating with **3b**. B) CD spectra of **DNA2** recorded when titrating with **3b**. C) CD spectra of **DNA4** recorded when titrating with **3b**. The direction of the arrows indicates the continuous changes with 0−25 µM **3b** at a specific λ. D) Structures of **DNA1**, **DNA2**, **DNA3**, and **DNA4**. Concentrations of **3b**: 0–25 µM. [DNA] = 5 µM.

To further confirm the triplex stabilization effect of 5‐substituted 2‐(naphthalen‐2‐yl)‐4*H*‐chromen‐4‐ones, we carried out thermal denaturation on **DNA1**, which forms a short intramolecular triplex DNA containing a 15 consecutive purine nucleotide triplex (GAAAAAAAAAAAAAG) forming region with two tetra‐thymidine loops (Figure 3D). Thermal denaturation of **DNA1** (1 µM/strand) showed a biphasic transition, with a T_m3→2_ value of 24.0°C and a T_m2→1_ value of 63.5°C. The ΔT_m_ values of **DNA1** in the presence of a ligand of interest (**1**, **3a**‐**3g**, **6**, **5b**, and **4b**) at 10 µM are shown in Figure 2C. A similar pattern can be observed compared with the results obtained for the polynucleotide triplex DNA, poly(dA)•2poly(dT). The parent compounds (**1** and **6**) lacking a side chain showed minimal triplex stabilization. A well‐separated biphasic transition was observed in both cases. Compounds **3a**‐**3g** showed excellent triplex‐stabilizing effects, as **DNA1** dissociated into random coils with a single‐phase transition. The ΔT_m3→1_ values of **DNA1** in the presence of **3a**‐**3g** are all between 41.1 and 45.0°C. Amongst all compounds (**3a**‐**3g**), compounds **3b** and **3** **g** are the most and least effective ligands to thermally stabilize triplex **DNA1**, respectively. In comparison, the melting profiles of **DNA1** in the presence of **5b** and **4b** are biphasic with a ΔT_m3→2_ value of (21.2 ± 0.1) °C and (12.0 ± 0.8) °C, respectively, further confirming that the size and curvature of the aromatic surface play a vital role in stacking with DNA triplets. A similar concentration‐dependent trend was observed on the melting profiles (Figure 2D) of **DNA1** to those of poly(dA)•2poly(dT), suggesting that the induced 3→1 transition by 5‐substituted 2‐(naphthalen‐2‐yl)‐4*H*‐chromen‐4‐ones is present in various DNA triplexes.

### 2.3 Binding of Triplex and Duplex DNA with 3b Determined by Circular Dichroism

CD measures the difference in absorption of left‐handed and right‐handed circularly polarized light when the light passes through chiral molecules. It has been used to determine the conformation of biomacromolecules such as proteins and nucleic acids [27, 28]. The binding of ligands to nucleic acids often accompanies conformational changes that can be detected by CD [29, 30, 31]. The magnitude of conformational changes usually reflects the strength of binding events, allowing semi‐quantitative comparison of binding between different ligands. In this study, we first carried out a titration experiment in which compound **3b** was titrated into a solution of **DNA1** (5 µM) in a sodium cacodylate buffer at pH 7.0. The temperature was maintained at 15°C to ensure the stability of the intramolecular triplex DNA in solution. As shown in Figure 3A, the CD spectrum of **DNA1** exhibits a positive peak at 278 nm, a small positive shoulder peak at 260 nm, and a negative peak at 247 nm, indicating the presence of a triplex DNA structure in solution [24]. Gradual addition of **3b** from 0 to 25 µM to the DNA solution leads to drastic conformational changes in the CD spectra of **DNA1**. The observations are listed as follows: a) Changes in five regions from 230 to 400 nm are present. The intensities of the peaks (maxima) at 247 nm, 278 nm, and 260 nm gradually decreased, while those at 316 nm and 360 nm gradually increased with increasing concentrations of **3b**. Furthermore, the negative peak at 247 nm shifted bathochromically to 253 nm. b) The intensity of the shoulder peak (maxima) at 260 nm significantly decreased with increasing concentrations of **3b**, which is consistent with the previously reported study on 5‐substituted flavones (compound **5b**) [24]. When the ligand concentration was above 10 µM, this shoulder peak almost disappeared completely. c) A clear isosbestic point was observed at 252 nm, suggesting the equilibrium between free and bound ligands in solution. d) The gradual increase of the negative peak at 316 nm and the positive peak at 360 nm represents the signals from bound **3b**. Free compound **3b** in solution is achiral; it should not induce a difference in left‐handed and right‐handed polarized light. Upon binding to triplex DNA, compound **3b** is restricted from its rotation in an asymmetric environment, leading to induced CD signals observed here. Interestingly, the magnitude of the changes in CD signals when titrating **3b** into **DNA1** is much more significant than titrating **5b** under the same conditions [24], suggesting that **3b** induces more triplex conformational changes than **5b**. This observation is a clear implication that **3b** binds to triplex DNA more strongly than **5b**, corroborating the conclusion from the thermal denaturation results mentioned above.

To compare the effect of **3b** on duplex DNA, we carried out another titration experiment with **DNA2** under the same conditions. **DNA2** is the portion of the duplex‐forming sequence of **DNA1** without the second tetra‐thymidine loop and any of the nucleotides after it (full sequence shown in Figure 3D). When titrating **3b** into **DNA2**, the spectra of **DNA2** (Figure 3B) showed noticeable but much less drastic changes compared with those of **DNA1** mentioned above. **DNA2** alone showed three major peaks: a negative peak at 248 nm, a slight positive peak at 260 nm, and a large positive peak at 282 nm. The intensities of the peaks (maxima) at 248 nm and 282 nm gradually decreased, while those at 315 nm and 360 nm gradually increased with increasing concentrations of **3b**. Overall, the shift of the maxima of each peak in this study is subtle. The intensity of the peak at 260 nm decreased with increasing concentrations of **3b**. Compared to data with **DNA1**, this peak never disappeared completely even when the concentration of **3b** was at 25 µM. A clear isosbestic point was observed at 253 nm. The gradual increases in the negative peak at 315 nm and the positive peak at 360 nm are much less intense than those observed in the experiments with **DNA1**, representing signals from a small amount of bound compound **3b**. It is worth noting that titrating **5b** into **DNA2** resulted in minimal conformational changes in **DNA2** because **5b** has weak‐to‐no interactions with duplex DNA [24].

Noticeable conformational changes upon titrating **3b** into duplex DNA (**DNA2**) are surprising and may result from the extreme potency of **3b** for binding to triplex DNA. By design, **DNA2** forms a hairpin‐like duplex structure; however, it could create an intermolecular triplex complex structure (**DNA3**, Figure 3D) under a condition (refer to the presence of **3b**) that strongly favors triplex formation. Compound **3b** could induce the formation of a small amount of **DNA3** structures in solution, leading to the observed conformational changes. To confirm this hypothesis, we designed **DNA4** (sequence shown in Figure 3D), which can form a hairpin‐like duplex but cannot form a triplex complex like **DNA3**. Titration of **3b** into a solution of **DNA4** showed no changes in the CD spectra of **DNA4** (Figure 3C), suggesting that **3b** could not interact with duplex DNA.

Taken together, we confirmed that **3b** interacts strongly with triplex DNA, while it has little effect on binding to duplex DNA.

### 2.4 Comparison of the Binding by the Fluorescent Intercalator Displacement Assay

The binding affinities of representative ligands (**1**, **3b**, and **3g)** to triplex DNA (**DNA1**) were further compared using the classic FID assay. Thiazole orange (**TO**), a cyanine dye that binds strongly to high‐order DNA structures, such as triplexes and G‐quadruplexes [32], is widely used in FID assays to evaluate ligand‐DNA interactions. This method provides a reliable and convenient means to compare relative ligand binding strengths [33].

**FIGURE 4:**
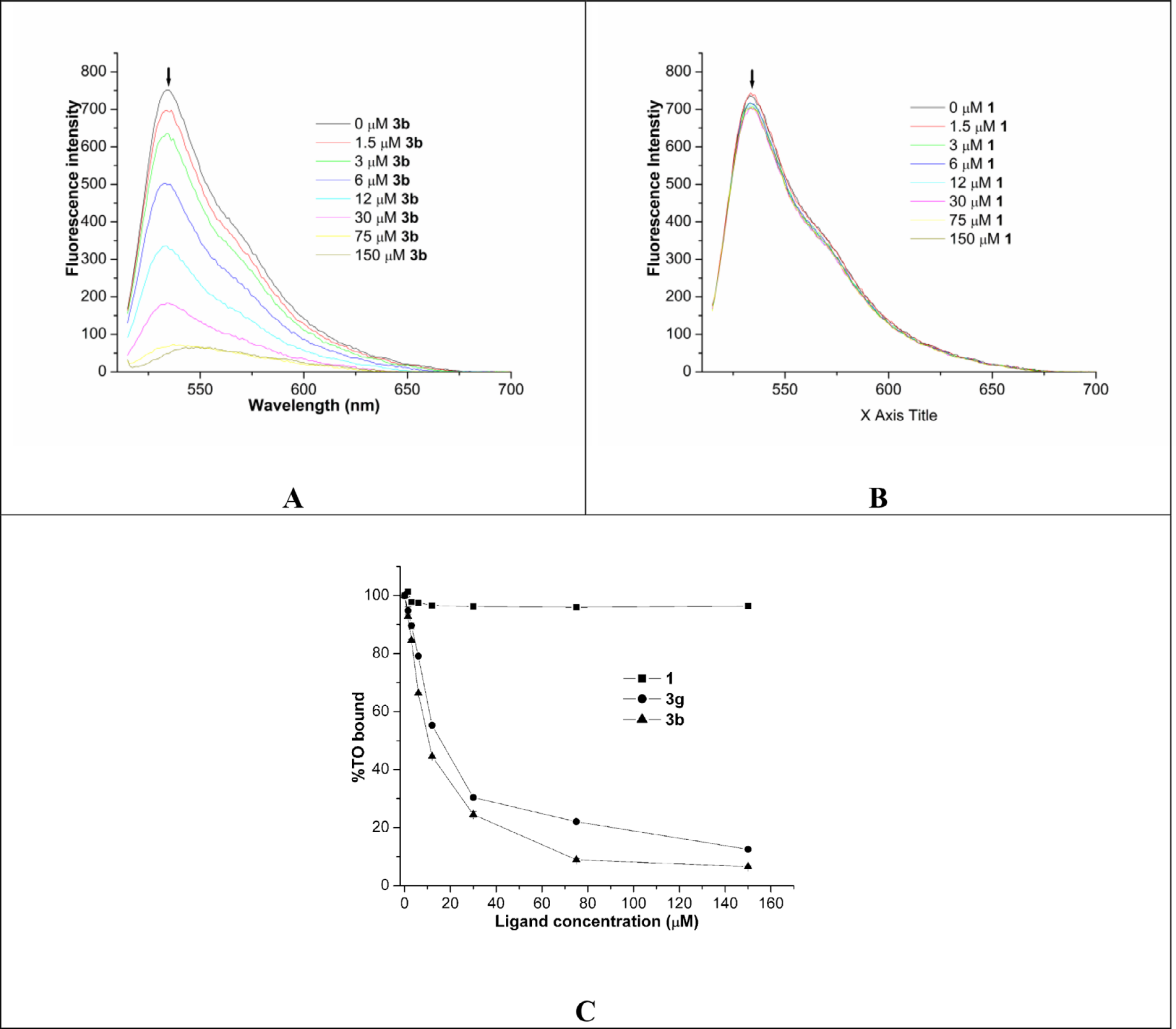
A) Fluorescence spectra of **TO**‐**DNA1** with addition of **3b**. B) Fluorescence spectra of **TO**‐**DNA1** with addition of **1**. C) %**TO** bound plotted as a function of increasing concentrations of **3b, 3g, and 1**. The direction of the arrows indicates the continuous changes with 0−150 µM ligand. Buffer: 10 mM sodium cacodylate (pH 7.0), 100 mM NaCl. Ex: 509 nm. [ligand] = 0−150 µM.

In this assay, **TO** (3 µM) was pre‐bound to **DNA1** (1.5 µM), emitting strong fluorescence (intensity = ∼750) with a λ_max_ of 533 nm (Figure 4A, B). Titration of **3b** (0‐150 µM) into the **TO**‐DNA mixture caused a sharp decrease in fluorescence. At 75 and 150 µM **3b**, the intensity fell below 70, a more than 10‐fold reduction (Figure 4A). A similar pattern was obtained when **3** **g** was titrated into the **TO**‐DNA mixture (data not shown). In contrast, titration of **1** produced only a minor reduction in fluorescence (Figure 4B), indicating poor displacement of **TO** and thus weak binding to triplex DNA **(DNA1**). This displacement event can be quantified using the DC_50_ value, which refers to the ligand concentration required to displace 50% of the pre‐bound **TO**. As shown in Figure 4C, the DC_50_ values of **3b** and **3** **g** to **DNA1** are 9.4 µM and 12.3 µM, respectively, suggesting that **3b** binds to triplex DNA slightly better than **3** **g**. The trend is consistent with the data obtained in the thermal denaturation experiments. No meaningful DC_50_ value could be determined for compound **1** within the concentration range. These results demonstrate that the pendant side chains in **3b** and **3** **g** are critical for effective binding to triplex DNA.

### 2.5 Determination of Binding Mode by Viscosity Studies

Upon confirming the strong interaction of **3b** with triplex DNA, we further investigated its binding mode using an established viscometric titration method in the lab. Determining the binding mode provides insight into ligand orientation and key contacts within the triplex binding pocket. Organic ligands typically bind to DNA via intercalation and groove binding, which can be differentiated by changes in DNA solution viscosity [34, 35]. Insertion of ligands between DNA base pairs (or triplets) leads to unwinding of DNA, elongation of the DNA helix, and, subsequently, an increase in solution viscosity. In an intercalation event, viscosity rises linearly with ligand concentration, giving a slope of 0.5 to 1 as a function of the ligand‐to‐DNA ratio (r_db_). In contrast, groove‐binding ligands do not alter the DNA strand's length and thus produce no viscosity changes.

**FIGURE 5:**
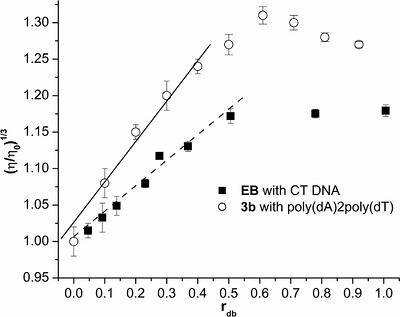
Viscosity changes of 0.5 mM poly(dA)•2poly(dT) (°) and 1 mM calf thymus DNA (▪) with increasing concentrations of **3b** and ethidium bromide (**EB**), ranging from 0−1 r_db_. Buffer: 10 mM sodium cacodylate (pH 7.0), 150 mM KCl. The error bars represent the standard deviation of three measurements.

In this viscosity study, viscosity measurements were performed using an Ostwald‐type viscometer. To validate the method, we first titrated ethidium bromide (**EB**), a well‐known intercalator, into a calf thymus (CT) DNA solution (1 mM in base pairs). The ligand‐to‐DNA ratio (r_db_) ranged from 0 to 1. As shown in Figure 5, the viscosity of the CT DNA solution increased proportionally (R^2^ = 0.98) with a slope of 0.35 over r_db_ ratios from 0 to 0.5. When the r_db_ ratios were above 0.5, an apparent plateau was observed, indicating saturation of intercalation sites in CT DNA. The inflection point at r_db_ = 0.5 corresponds to one **EB** molecule per two DNA base pairs, consistent with the nearest neighbor‐exclusion model [36]. When titrating **3b** into the polynucleotide triplex [poly(dA)•2poly(dT)], we also observed a drastic increase in solution viscosity over r_db_ ratios from 0 to 0.5. Notably, the concentration of poly(dA)•2poly(dT) used here was 0.5 mM (in base triplets) due to material cost. Within the same r_db_ range, **3b** induced a markedly greater viscosity increase than EB with CT DNA, implying the stronger affinity of **3b** with triplex DNA. A linear fit (R^2^ = 0.98) between r_db_ = 0‐0.4 yielded a slope of 0.6, characteristic of intercalative binding. Beyond r_db_ = 0.4, white flocculent precipitates formed, prohibiting accurate determination of the saturation r_db_ value; therefore, the inflection point was not pursued.

### 2.6 Visualization of Ligand‐Induced Triplex Formation via PAGE

The electrophoretic mobility of single‐stranded, duplex, and triplex DNA differs on nondenaturing polyacrylamide gels due to variations in size and volume. Accordingly, gel electrophoretic mobility shift assays are widely used to distinguish DNA secondary structures.

**FIGURE 6:**
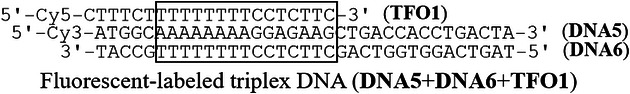
The sequences of **DNA5**, **DNA6**, and **TFO1** in the gel mobility shift assay. The triplex‐forming region is highlighted in the box.

In this experiment, we conducted such an assay to visually monitor the formation of triplex DNA induced by **3b** across ligand concentrations of 0–20 µM. This assay comprised three DNA oligonucleotides, including a Cy3‐labeled oligonucleotide **DNA5**, an unlabeled complementary strand **DNA6**, and a Cy5‐labeled triplex‐forming oligonucleotide **TFO1** (Figure 6). Annealing **DNA5** and **DNA6** forms a Cy3‐tagged DNA duplex. Combining all three strands under appropriate conditions produces the triplex (**DNA5**:**DNA6**•**TFO**). We ran the gel in Tris‐acetate buffer at pH 5.0, which favors C^+^•G:C triplet formation.

**FIGURE 7:**
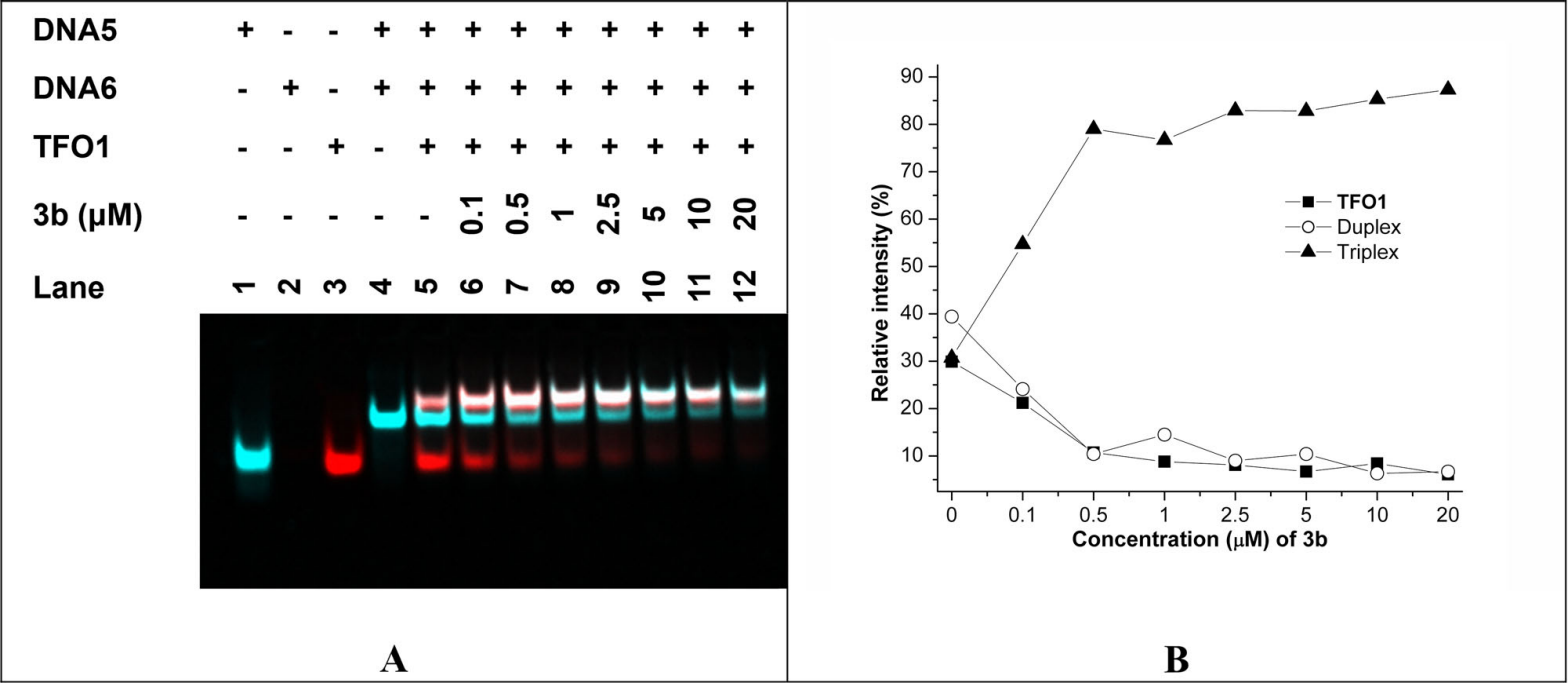
A) The gel mobility shift assay shows the formation of triplex DNA (formed by **DNA5**, **DNA6**, and **TFO1**) as a function of increasing concentrations of **3b**. Buffer: 90 mM tris‐acetate, pH 5.0. B) Changes in relative intensity (%) of the duplex, **TFO1**, and triplex bands (in the presence of **3b** at various concentrations ranging from 0 to 20 µM.

Gel images monitored by fluorescence are shown in Figure 7A. **DNA5** (lane 1) and **TFO1** (lane 3) appear as cyan and red bands, respectively, with similar mobilities due to their comparable lengths. Unlabeled **DNA6** (lane 2) is undetectable. Note: our gel imager cannot accurately display the true colors of fluorophores; therefore, Cy3 and Cy5 are shown in cyan and red, respectively, for clarity. Mixing equimolar amounts of **DNA5 and DNA6** (lane 4) yields a slower‐moving cyan duplex band. Mixing **DNA5**, **DNA6**, and **TFO1** (lane 5) generates a new whitish band (with some red background) that migrates more slowly than the duplex band, diagnostic of triplex assembly. Residual duplex and **TFO1** bands in lane 5 indicate incomplete triplex formation, consistent with moderate triplex stability in solution. Lane 5 serves as the reference (positive control) for lanes 6−12.

Addition of **3b** (0.1−20 µM, lanes 6−12) progressively depletes the duplex (cyan) and **TFO1** (red) while intensifying the whitish triplex band. Relative fluorescence intensities are quantified as follows. Densitometric values for each band (lanes 5–12) were obtained from the Cy5 and Cy3 channels. Triplex bands appeared in both channels, so their values were summed. Duplex and **TFO1** bands were detected exclusively in the Cy3 and Cy5 channels, respectively. The relative intensity (%) of each species was then calculated as the ratio of its total densitometric value to the sum of the three bands (Figure 7B).

As the **3b** concentration increased (0.1‐20 µM), the relative intensity (%) of **TFO1** and duplex decreased steadily, while that of the triplex increased gradually. In the absence of ligand (lane 5), the values were 30.7% (triplex), 39.5% (duplex), and 29.9% (**TFO1**). Triplex induction became detectable at 0.1 µM **3b**, where triplex intensity rose to 54.7%, with duplex and **TFO1** falling to 24.1% and 21.2%, respectively. At 0.5 µM **3b**, triplex intensity reached 79%, and both duplex and **TFO1** dropped to ∼10%. All bands plateaued thereafter. By contrast, our previous study revealed that **5b** at 20 µM reduced these bands only to ∼40% [24], confirming that **3b** is superior in triplex‐stabilizing efficiency.

As shown here, triplex intensity increased with increasing concentrations of **3b**. However, densitometric analysis could be skewed by heterogeneous fluorescence emission or by enhanced emission upon **3b** binding to triplex DNA (described in the next section). Despite these potential artifacts, the trends (Figure 7B) clearly demonstrate that **3b** induces triplex formation effectively even at submicromolar concentrations.

### 2.7 Binding of 3b with Triplex DNA Induces Fluorescence Visible Under UV Illumination

**FIGURE 8:**
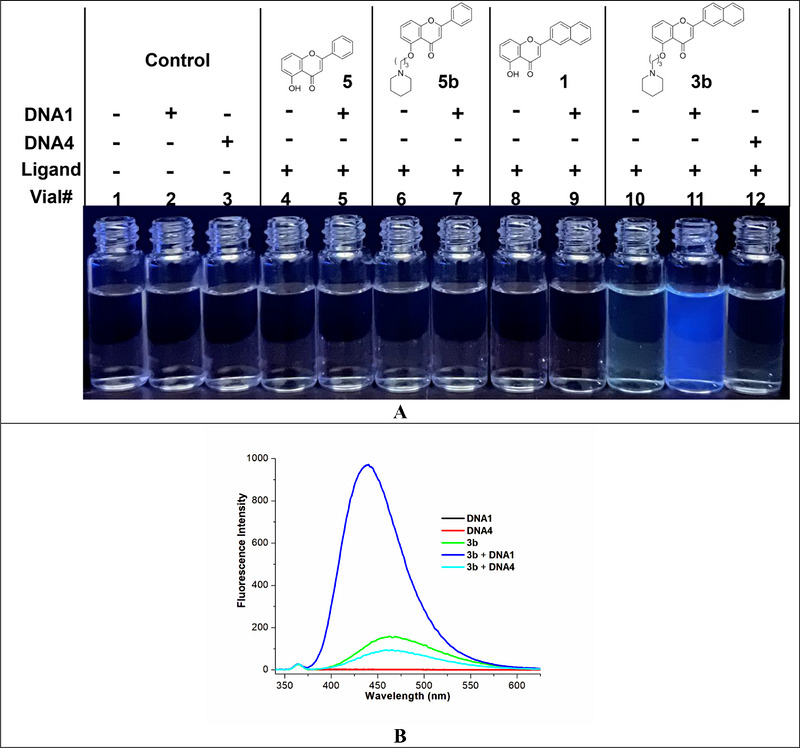
A) Images of **DNA1**, **DNA4**, and ligand (**5**, **5b**, **1**, and **3b**) solutions under UV illumination. [DNA] = 1 µM/strand and [ligand] = 10 µM. B) Fluorescence spectra of **DNA1**, **DNA4**, **3b**, **3b** + **DNA1**, and **3b** + **DNA4**. Buffer: 10 mM sodium cacodylate, 100 mM NaCl, pH 7.0.

The extended aromatic surface of **3b** may restrict rotational freedom upon intercalation, thereby enhancing conjugation and fluorescence. In this study, we examined the fluorescence emission properties of **5**, **5b**, **1**, and **3b** (10 µM) in the presence of triplex **DNA1** (1 µM) or duplex **DNA4** (1 µM) in glass vials (Figure 8A).

None of the control samples (vials 1–3) showed any fluorescence responses. Compound **3b** displayed intense blue fluorescence when incubated with **DNA1** (triplex DNA) in sodium cacodylate buffer (10 mM) at pH 7.0 (vial 11), readily visible to the naked eye under UV illumination. Compound **3b** alone showed only a faint greenish color (vial 10), confirming that the observed strong blue fluorescence activation requires triplex binding. Compound **3b** did not emit the same strong blue fluorescence upon incubation with **DNA4** (vial 12), a sequence that can only form a hairpin‐like duplex, indicating that **3b** could not effectively interact with duplex DNA. In contrast, compounds **1**, **5**, and **5b** exhibited negligible fluorescence emission even in the presence of **DNA1** under the same conditions (vials 4–9).

Five selected solutions (**DNA1**, **DNA4**, compound **3b**, **3b** + **DNA1**, and **3b** + **DNA4**) were further diluted fivefold with the same buffer. Fluorescence spectra of the resulting solutions were recorded between 340 and 625 nm using an excitation wavelength of 323 nm. As shown in Figure 8B, neither **DNA1** nor **DNA4** exhibited any detectable fluorescence under these conditions, as anticipated. Compound **3b** alone displayed weak fluorescence with an emission maximum (λ_max_) at 465 nm. When incubated with **DNA4** (duplex DNA), the fluorescence spectrum of **3b** remained essentially unchanged, though the intensity slightly decreased. In contrast, binding of **3b** to triplex DNA (**DNA1**) led to a dramatic ∼eightfold increment in fluorescence intensity, accompanied by a clear hypsochromic shift of the λ_max_ to 440 nm. These quantitative fluorescence changes were in good agreement with the visual images under UV illumination.

This selective “light‐up” response makes **3b** a promising fluorogenic probe for detecting triplex DNA in solution, which warrants further investigation. Fluorogenic probes for the detection of noncanonical DNA structures have long been a focus of research. Brown and coworkers reported thiazole‐orange‐based probes for the detection of parallel DNA triplexes at neutral pH and above [37].

### 2.8 Inhibition of Restriction Enzyme Activity via Ligand‐Mediated Triplex Formation

Noncanonical triplex structures in disease‐related genes can inhibit or regulate biological processes. Rajeswari and coworkers reported that DNA triplex formation inhibits MET expression, inducing cell death and tumor regression in hepatoma [38]. Mattick and Bailey's team reviewed [39] the potential in vivo roles of nucleic acid triple helices in chromatin organization [40], DNA repair [41] and damage [42], transcriptional regulation [43, 44], and gene silencing [45]. Antigen enhancers should further enhance these biological processes.

**FIGURE 9:**
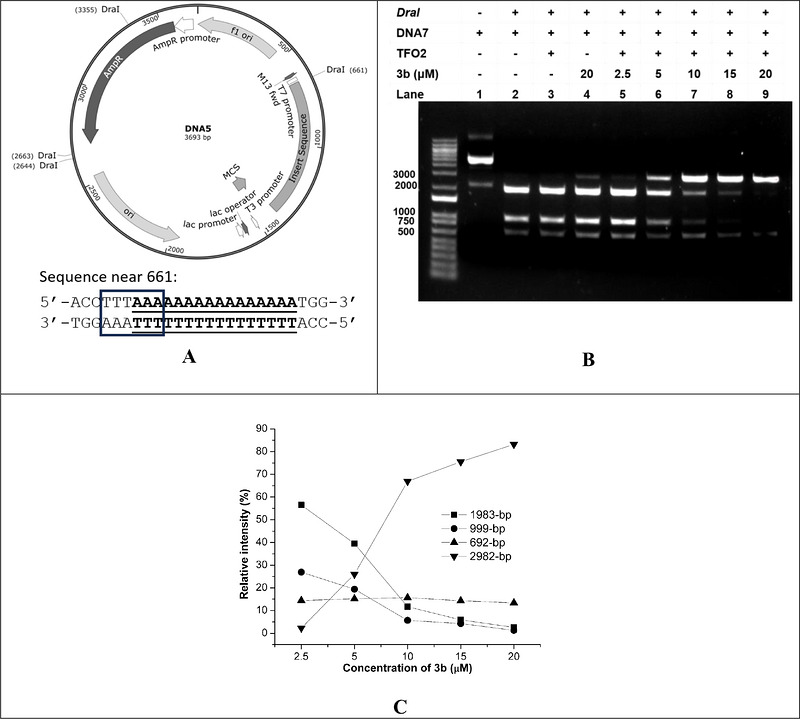
A) The constructed 3693‐bp plasmid (**DNA7**) contains four *DraI* cleavage sites at positions 661, 2644, 2663, and 3355. The DNA sequence near position 661 contains a 16‐mer deoxyadenosine‐thymidine sequence as the triplex‐forming region (labeled in bold underlined text). The *DraI* cleavage site at position 661 is indicated in the box. B) Agarose gel shows the cleavage of the plasmid (**DNA7**) by *DraI* under various conditions as indicated. **TFO2**: a 16‐mer oligothymidine strand. C) Changes in relative intensity (%) of four cleaved fragments (692‐bp, 999‐bp, 1983‐bp, and 2982‐bp) in the presence of **3b** (0 to 20 µM).

Our lab previously established a plasmid‐based assay to quantify in vitro inhibition of enzymatic activity via ligand‐mediated triplex formation [24]. In this assay, a modified plasmid DNA (**DNA7**) (Figure 9A; full sequence shown in Figure S1 of the supporting information) derived from pBluescript II SK(+) contains four *DraI* restriction sites. One site overlaps a 16‐bp oligopurine‐oligopyrimidine (AT‐rich) triplex‐forming sequence near the T7 promoter. Digestion of **DNA7** with *DraI* yields four fragments: 19 bp, 692 bp, 999 bp, and 1983 bp. When a 16‐mer oligothymidine (**TFO2**) binds to the target duplex, it blocks *DraI* at that site, preventing cleavage and producing a new 2982‐bp fragment (999 bp + 1983 bp) while eliminating the 999‐bp and 1983‐bp bands. Coadministration of the TFO (**TFO2**) and a triplex‐stabilizing ligand enhances the inhibition of *DraI*. Digested DNA fragments are separated by agarose gel electrophoresis and visualized with ethidium bromide.

Results for **DNA7** digested by *DraI* under various conditions are shown in Figure 9B. The 19‐bp fragment is too small to be retained on the gel. A DNA ladder (leftmost lane) provides a size marker. Lane 1 shows undigested plasmid **DNA7** (negative control). Lane 2 shows the digestion of **DNA7** by *DraI* with three visible fragments (692 bp, 999 bp, and 1983 bp), confirming complete cleavage and loss of supercoiled plasmid. Lane 3 represents the digestion of **DNA7** by *DraI* in the presence of **TFO2**. Lane 3 reproduces the same pattern as lane 2, indicating that the 16‐mer **TFO2** fails to bind efficiently under these conditions, consistent with its T_m3→2_< 10°C. Lane 4 represents the digestion of **DNA7** by *DraI* in the presence of 20 µM **3b**. It shows the same three major fragments, but a faint ∼2982‐bp band appears. This faint band arises from plausible **3b**‐induced triplex formation at the 16‐bp AT site by shorter (6‐7 bp) AT‐tract sequences elsewhere in the plasmid that partially block *DraI*. Lanes 5–9 represent the digestion of **DNA7** by *DraI* in the presence of **TFO2** as a function of increasing **3b** concentrations (2.5−20 µM). The ∼3000‐bp band gradually intensifies, while the 999‐bp and 198‐bp bands diminish in a concentration‐dependent manner. Notably, the 692‐bp band remains the same (unaffected site). At 2.5 µM **3b** (lane 5), the 2982‐bp fragment is faint. At 5 µM **3b**, it becomes prominent (lane 6). And at 20 µM **3b** (lane 9), the 999‐bp and 1983‐bp bands nearly vanish, leaving only the 2982‐bp and 692‐bp fragments. In contrast, our previous studies reported that the digestion of **DNA7** by *DraI* in the presence of the **TFO2** requires at least 100 µM **5b** to achieve a detectable 2982‐bp fragment. It is worth noting that the intensities of the 2982‐bp fragment in lanes 4 and 9 differ markedly, indicating that the observed inhibition of *DraI* activity in lane 9 must result from a synergistic effect of both **TFO2** and **3b**. The gel results can be quantified using a detailed densitometric analysis of each band (lanes 5−9), shown in Figure 9C. The relative intensity (%) of each band was calculated as the ratio of its total densitometric value to the sum of densitometric values of all four bands. From 2.5 to 20 µM **3b**, the relative intensity (%) of the 2982‐bp fragment gradually increased from 2.3% to 83.2%, while the relative intensity (%) of the 1983‐bp and 999‐bp fragments drastically reduced from 56.5% to 2.6% and 26.9% to 1.3%, respectively. Notably, the relative intensity (%) of the 692‐bp fragment remained almost unchanged (∼15%) across this concentration range.

These results demonstrate that **3b**, one of the most potent triplex‐specific ligands from our laboratory, effectively inhibits *DraI* activity via ligand‐mediated triplex formation at low micromolar concentrations. This capability paves the way for investigating transcriptional regulation in cells by **3b**‐mediated triplex formation, with results to be reported in due course. Such ligands hold strong promise as antigen enhancers for regulating diverse enzymatic processes.

## 3 Conclusion

Compared to the previously reported 5‐substituted flavone (**5b**), compound **3b** exhibits superior performance across all biophysical studies, demonstrating that expanding the aromatic surface area is an effective strategy for enhancing triplex DNA binding. UV thermal denaturation studies reveal that 5‐substituted 2‐(naphthalen‐2‐yl)‐4*H*‐chromen‐4‐ones promote triplex DNA to dissociate directly into random coils, bypassing a duplex intermediate, a hallmark of potent triplex ligands. Viscosity measurements confirm that these ligands bind to triplex DNA via intercalation. Multiple biophysical techniques, including UV thermal denaturation, CD, and FID assays, consistently show that 5‐substituted 2‐(naphthalen‐2‐yl)‐4*H*‐chromen‐4‐ones exhibit strong selectivity for triplex DNA over duplex DNA. The presence of an amino‐containing side chain at the 5‐position proves crucial for both binding selectivity and affinity. Notably, compound **3b** emits intense blue fluorescence upon binding to triplex DNA, a phenomenon absent with duplex DNA, providing further evidence of its selective interaction with triplex DNA. Importantly, the representative compound **3b** can promote triplex formation at submicromolar concentrations and inhibit restriction endonuclease activity via ligand‐mediated triplex formation at low micromolar concentrations.

Collectively, the studies presented here establish the 5‐substituted 2‐(naphthalen‐2‐yl)‐4*H*‐chromen‐4‐one scaffold as a promising platform for developing triplex‐selective agents. Key design principles emerging from our recent work could include: 1) Amino‐containing pendant chains at the 5‐position that occupy the triplex groove(s) are essential for achieving high affinity and selectivity. 2) Expansion of the aromatic system in ring B enhances π‐π stacking with base triplets and better accommodates the propeller twist of base triplets, leading to significantly improved binding strength. Overall, potent triplex‐binding ligands such as **3b** represent promising candidates for antigene applications in future gene‐regulation studies.

## 4 Experimental

### 4.1 Materials and General Methods

All chemicals were purchased from MilliporeSigma or Fisher Scientific and used without further purification. DNA oligonucleotides were purchased from Fisher Oligos and Integrated DNA Technologies. Polynucleotides were purchased from MilliporeSigma. The concentrations of polynucleotides were determined spectrophotometrically using the following extinction coefficients (in units of mol of nucleotide/L^−1^ cm^−1^): ε_260_ = 6,000 for poly(dA)•poly(dT), ε_265_ = 9,000 for poly(dT), and ε_260_ = 6,600 for calf thymus (CT) DNA. The concentrations of oligonucleotides were determined spectrophotometrically using the following extinction coefficients (in units of mol of strand/L^−1^ cm^−1^) obtained from OligoAnalyzer (www.idtdna.com): ε_260_ = 483,700 for **DNA1** (5′‐GAAAAAAAAAAAAAGTTTTCTTTTTTTTTTTTTCTTTTCTTTTTTTTTTTTTC‐3′), ε_260_ = 331,600 for **DNA2** (5′‐GAAAAAAAAAAAAAGTTTTCTTTTTTTTTTTTTC‐3′), ε_260_ = 327,400 for **DNA4** (5′‐GATCTACCACATCTGTTTTCAGATGTGGTAGATC‐3′), ε_260_ = 368,100 for **DNA5** (5′‐/Cy3/ATGGCAAAAAAAAGGAGAAGCTGACCACCTGACTA‐3′), ε_260_ = 312,100 for **DNA6** (5′‐TAGTCAGGTGGTCAGCTTCTCCTTTTTTTTGCCAT‐3′), ε_260_ = 174,900 for **TFO1** (5′‐/Cy5/CTTTCTTTTTTTTTCCTCTTC‐3′), and ε_260_ = 130,200 for **TFO2** (5′‐TTTTTTTTTTTTTTTT‐3′). UV spectra were recorded on a Varian Cary‐100 Bio UV‐Vis spectrophotometer equipped with a thermoelectrically controlled 6×6 cell holder. CD spectra were recorded on a JASCO J‐810 spectropolarimeter using a quartz cuvette with a 1 cm optical path length. Fluorescence spectra were recorded on a PerkinElmer LS‐55 fluorescence spectrometer. IR spectra were collected on a Thermo Scientific Nicolet iS10 FTIR spectrometer. Gel images were visualized on an Invitrogen iBright FL1500 imaging system. ^1^H‐NMR and ^13^C‐NMR spectra were recorded on a JEOL ECA 600 MHz FT‐NMR spectrometer. MS spectra for small molecules were recorded on an Agilent LC‐qTOF with electrospray ionization (ESI). The plasmid DNA (**DNA7**) was designed using SnapGene (Boston, MA, USA) and synthesized by GenScript Biotech (Piscataway, NJ, USA). Viscosity data were collected using an Ostwald‐type viscometer.

### 4.2 Synthesis

#### 4.2.1 Synthesis of 5‐Hydroxy‐2‐(Naphthalen‐2‐yl)‐4*H*‐Chromen‐4‐One (1)

In a round‐bottomed flask, 2′, 6′‐dihydroxyacetophenone (500 mg, 3.29 mmol) was dissolved in 20 mL of acetone, followed by addition of potassium carbonate (2.27 g, 16.4 mmol, 5 equiv.). The reaction was stirred at room temperature for 10 min. After addition of 2‐naphthoyl chloride (0.626 g, 3.29 mmol, 1 equiv.), the reaction mixture was refluxed at 75°C for 5 h. The reaction was monitored using TLC (R_f_ = 0.22, hexane/ethyl acetate = 95:5 v/v, HPTLC Silica Gel 60 GF_254_) by UV. The reaction mixture was concentrated under vacuum and then partitioned between ethyl acetate (100 mL) and water (20 mL). The organic layer was then washed with water (20 mL x 2) brine (20 mL), dried with anhydrous sodium sulfate, and concentrated under vacuum. Flash column chromatography of the residue (silica gel, hexane/ethyl acetate = 95:5 v/v) yielded compound **1** (214 mg, 23.0%) as a yellow solid. IR (KBr, cm^−1^): 3068, 2917, 1650, 1612, 1440, 823, 746. ^1^H‐NMR (600 MHz, CDCl_3_): δ 8.47 (s, 1H), 7.99‐7.96 (m, 2H), 7.90‐7.89 (m, 2H), 7.63‐7.56 (m, 3H), 7.08 (d, *J* = 7.2 Hz, 1H), 6.86 (s, 1H), 6.83 (d, *J* = 8.4 Hz, 1H). ^13^C‐NMR (150 MHz, CDCl_3_): δ 183.7, 164.6, 160.9, 156.6, 135.5, 134.9, 132.9, 129.2, 129.1, 128.42, 128.38, 127.9, 127.4, 127.3, 122.5, 111.6, 111.0, 107.2, 106.4. HRMS (ESI) *m/z*: calcd. for C_19_H_12_O_3_ [M+H]^+^: 289.0859, found 289.0883.

#### 4.2.2 Synthesis of 5‐(3‐Bromopropoxy)‐2‐(Naphthalen‐2‐yl)‐4*H*‐Chromen‐4‐One (2)

In a small round‐bottomed flask, compound **1** (481 mg, 1.67 mmol) was dissolved in 8 mL of DMF at 90°C and cooled down to room temperature followed by potassium carbonate (0.692 g, 5.01 mmol, 3 equiv.). After addition of 1,3‐dibromopropane (1.53 mL, 15.0 mmol, 9 equiv.), the reaction mixture was stirred at room temperature for 4 days. The reaction was monitored using TLC (R_f_ = 0.27, hexane/ethyl acetate = 80:20 v/v, HPTLC Silica Gel 60 GF_254_) by UV. The reaction mixture was concentrated under vacuum and then partitioned between ethyl acetate (50 mL) and water (5 mL). The organic layer was washed with water (5 mL x 2), brine (5 mL), dried with anhydrous sodium sulfate, and concentrated under vacuum. Flash column chromatography of the residue (silica gel, hexane/ethyl acetate = 75/25 v/v) yielded compound **2** (302 mg, 44.2%) as a white solid. IR (KBr, cm^−1^): 2922, 1601, 1379, 1094, 753. ^1^H‐NMR (600 MHz, CDCl_3_): δ 8.42 (s, 1H), 7.96‐7.92 (m, 2H), 7.88‐7.86 (m, 2H), 7.60‐7.54 (m, 3H), 7.20 (d, *J* = 8.4 Hz, 1H), 6.88 (s, 1H), 6.84 (d, *J* = 8.4 Hz, 1H), 4.25 (t, *J* = 5.4 Hz, 2H), 3.87 (t, *J* = 6.3, 2H), 2.45 (quint*, J* = 6.0 Hz, 2H).^13^C‐NMR (150 MHz, CDCl_3_): δ 178.2, 161.5, 159.1, 158.4, 134.7, 134.0, 133.0, 129.1, 129.0, 128.6, 128.0, 127.9, 127.1, 126.8, 122.5, 114.8, 110.5, 109.2, 108.0, 66.7, 32.2, 30.8. HRMS (ESI) *m/z*: calcd. for C_22_H_17_BrO_3_ [M+H]^+^: 409.0434, found 409.0438.

#### 4.2.3 Synthesis of 2‐(Naphthalen‐2‐yl)‐5‐(3‐(Pyrrolidin‐1‐yl)Propoxy)‐4*H*‐Chromen‐4‐One (3a)

In a round‐bottomed flask, compound **2** (160 mg, 0.391 mmol) was added in 8 mL of DMF and gently heated until it was fully dissolved followed by addition of potassium carbonate (0.162 g, 1.17 mmol, 3 equiv.) and pyrrolidine (196 µL, 2.38 mmol, 6 equiv.). The reaction mixture was stirred at room temperature for 20 h. The reaction was monitored using TLC (R_f_ = 0.25, DCM/methanol = 90:10 v/v, HPTLC Silica Gel 60 GF_254_) by UV. The reaction mixture was concentrated under vacuum and then partitioned between ethyl acetate (40 mL) and water (5 mL). The organic layer was washed with water (5 mL), brine (5 mL), dried with anhydrous sodium sulfate, and concentrated under vacuum. Flash column chromatography of the residue (silica gel, DCM/methanol = 95/5 v/v with 0.5‐1% v/v of 28–30% ammonium hydroxide) yielded compound **3a** (150 mg, 96.0%) as an off‐white solid. IR (KBr, cm^−1^): 2963, 1636, 1603, 1458, 1267, 1092, 746. ^1^H‐NMR (600 MHz, CDCl_3_): δ 8.38 (s, 1H), 7.93‐7.89 (m, 2H), 7.85‐7.81 (m, 2H), 7.59‐7.52 (m, 3H), 7.18 (d, *J* = 8.4 Hz, 1H), 6.80 (d, *J* = 8.4 Hz, 1H), 6.74 (s, 1H), 4.22 (t, *J* = 6.0 Hz, 2H), 3.43 (t, *J* = 6.9 Hz, 2H), 3.28‐3.15 (m, 4H), 2.37 (quint, *J* = 6.0 Hz, 2H), 2.14‐2.04 (m, 4H).^13^C‐NMR (150 MHz, CDCl_3_): δ 178.7, 161.7, 158.30, 158.28, 134.7, 134.3, 132.9, 129.1, 129.0, 128.3, 128.1, 127.9, 127.2, 126.8, 122.4, 114.4, 110.8, 109.1, 107.8, 67.5, 54.4, 53.8, 26.5, 23.6. HRMS (ESI) *m/z*: calcd. for C_26_H_25_NO_3_ [M+H]^+^: 400.1907, found 400.1920.

#### 4.2.4 Synthesis of 2‐(Naphthalen‐2‐yl)‐5‐(3‐(Piperidin‐1‐yl)Propoxy)‐4*H*‐Chromen‐4‐One (3b)

In a round‐bottomed flask, compound **2** (120 mg, 0.293 mmol) was added in 8 mL of DMF and gently heated until it was fully dissolved followed by addition of potassium carbonate (0.122 g, 0.883 mmol, 3 equiv.) and piperidine (174 µL, 1.76 mmol, 6 equiv.). The reaction mixture was stirred at room temperature for 24 h. The reaction was monitored using TLC (R_f_ = 0.30, DCM/methanol = 90:10 v/v, HPTLC Silica Gel 60 GF_254_) by UV. The reaction mixture was concentrated under vacuum and then partitioned between ethyl acetate (40 mL) and water (5 mL). The organic layer was washed with water (5 mL), brine (5 mL), dried with anhydrous sodium sulfate, and concentrated under vacuum. Flash column chromatography of the residue (silica gel, DCM/methanol = 95/5 v/v with 0.5‐1% v/v of 28–30% ammonium hydroxide) yielded compound **3b** (68.0 mg, 56.1%) as an off‐white solid. IR (KBr, cm^−1^): 2934, 1649, 1602, 1384, 1268, 1094, 747. ^1^H‐NMR (600 MHz, CDCl_3_): δ 8.35 (s, 1H), 7.91‐7.87 (m, 2H), 7.83‐7.78 (m, 2H), 7.57‐7.50 (m, 3H), 7.17 (d, *J* = 8.4 Hz, 1H), 6.78 (d, *J* = 8.4 Hz, 1H), 6.72 (s, 1H), 4.19 (t, *J* = 5.4 Hz, 2H), 3.50 (t, *J* = 7.2 Hz, 2H), 3.41‐2.97 (m, 4H), 2.48 (quint, *J* = 6.0 Hz, 2H), 2.12‐1.92 (m, 4H), 1.74‐1.58 (m, 2H). ^13^C‐NMR (150 MHz, CDCl_3_): δ 178.5, 161.8, 158.20, 158.18, 134.7, 134.4, 132.9, 129.1, 129.0, 128.2, 128.1, 127.9, 127.2, 126.8, 122.3, 114.4, 110.9, 109.0, 108.0, 67.2, 55.9, 53.8, 23.8, 22.7, 22.2. HRMS (ESI) *m/z*: calcd. for C_27_H_27_NO_3_ [M+H]^+^: 414.2064, found 414.2074.

#### 4.2.5 Synthesis of 5‐(3‐Morpholinopropoxy)‐2‐(Naphthalen‐2‐yl)‐4*H*‐Chromen‐4‐One (3c)

In a round‐bottomed flask, compound **2** (191 mg, 0.467 mmol) was added in 8 mL of DMF and gently heated until it was fully dissolved followed by addition of potassium carbonate (0.194 g, 1.40 mmol, 3 equiv.) and morpholine (242 µL, 2.81 mmol, 6 equiv.). The reaction mixture was stirred at room temperature for 16 h. The reaction was monitored using TLC (R_f_ = 0.43, DCM/methanol = 90:10 v/v, HPTLC Silica Gel 60 GF_254_) by UV. The reaction mixture was concentrated under vacuum and then partitioned between ethyl acetate (40 mL) and water (5 mL). The organic layer was washed with water (5 mL), brine (5 mL), dried with anhydrous sodium sulfate, and concentrated under vacuum. Flash column chromatography of the residue (silica gel, DCM/methanol = 95/5 v/v with 0.5‐1% v/v of 28–30% ammonium hydroxide) yielded compound **3c** (184 mg, 94.8%) as an off‐white solid. IR (KBr, cm^−1^): 2956, 2774, 1639, 1601, 1386, 1095, 862, 754. ^1^H‐NMR (600 MHz, CDCl_3_): δ 8.39 (s, 1H), 7.94‐7.89 (m, 2H), 7.85‐7.82 (m, 2H), 7.56‐7.52 (m, 3H), 7.17 (d, *J =* 8.4 Hz, 1H), 6.80 (d, *J* = 8.4 Hz, 1H), 6.77 (s, 1H), 4.19 (t, *J* = 6.0 Hz, 2H), 3.90‐3.78 (m, 4H), 3.02‐2.88 (m, 2H), 2.84‐2.68 (m, 4H), 2.23 (quint, *J* = 6.0 Hz, 2H). ^13^C‐NMR (150 MHz, CDCl_3_): δ 178.3, 161.2, 159.1, 158.4, 134.6, 133.9, 133.0, 129.1, 128.9, 128.6, 128.0, 127.9, 127.1, 126.7, 122.5, 114.9, 110.3, 109.4, 107.9, 67.6, 66.5, 55.6, 53.6, 25.7. HRMS (ESI) *m/z*: calcd. for C_26_H_25_NO_4_ [M+H]^+^: 416.1856, found 416.1876.

#### 4.2.6 Synthesis of 5‐(3‐(4‐Methylpiperazin‐1‐yl)Propoxy)‐2‐(Naphthalen‐2‐yl)‐4*H*‐Chromen‐4‐One (3d)

In a round‐bottomed flask, compound **2** (158 mg, 0.386 mmol) was added in 8 mL of DMF and gently heated until it was fully dissolved followed by addition of potassium carbonate (0.160 g, 1.16 mmol, 3 equiv.) and 1‐methylpiperazine (257 µL, 2.31 mmol, 6 equiv.). The reaction mixture was stirred at room temperature for 20 h. The reaction was monitored using TLC (R_f_ = 0.1, DCM/methanol = 90:10 v/v, HPTLC Silica Gel 60 GF_254_) by UV. The reaction mixture was concentrated under vacuum and then partitioned between ethyl acetate (40 mL) and water (5 mL). The organic layer was washed with water (10 mL x 2), brine (5 mL), dried with anhydrous sodium sulfate, and concentrated under vacuum. Flash column chromatography of the residue (silica gel, DCM/methanol = 90/10 v/v with 0.5‐1% v/v of 28–30% ammonium hydroxide) yielded compound **3d** (153 mg, 92.5%) as an off‐white solid. IR (KBr, cm^−1^): 2779, 1642, 1383, 1268, 1094, 746. ^1^H‐NMR (600 MHz, CDCl_3_): δ 8.35 (s, 1H), 7.91‐7.89 (m, 1H), 7.88‐7.86 (m, 1H), 7.81 (td, *J* = 7.5, 2.0 Hz, 2H), 7.54‐7.49 (m, 3H), 7.13 (dd, *J* = 8.4, 0.6 Hz, 1H), 6.78 (d, *J* = 8.4 Hz, 1H), 6.75 (s, 1H), 4.14 (t, *J* = 6.3 Hz, 2H), 2.69 (t, *J* = 7.2 Hz, 2H), 2.68‐2.31 (m, 8H), 2.28 (s, 3H), 2.10 (quint, *J* = 6.0 Hz, 2H). ^13^C‐NMR (150 MHz, CDCl_3_): δ 178.2, 161.0, 159.2, 158.4, 134.6, 133.8, 132.9, 129.0, 128.9, 128.7, 127.90, 127.85, 127.0, 126.6, 122.5, 114.9, 110.2, 109.4, 107.9, 67.7, 54.9, 52.9, 45.9, 26.3. HRMS (ESI) *m/z*: calcd. for C_27_H_28_N_2_O_3_ [M+H]^+^: 429.2173, found 429.2181.

#### 4.2.7 Synthesis of 5‐(3‐(Dimethylamino)Propoxy)‐2‐(Naphthalen‐2‐yl)‐4*H*‐Chromen‐4‐One (3e)

In a round‐bottomed flask, compound **2** (115 mg, 0.281 mmol) was added in 8 mL of DMF and gently heated until it was fully dissolved followed by addition of potassium carbonate (0.117 g, 0.847 mmol, 3 equiv.) and dimethylamine (2 M in THF, 843 µL, 6 equiv.). The reaction mixture was stirred at room temperature for 2 days. The reaction was monitored using TLC (R_f_ = 0.40, DCM/methanol = 90:10 v/v, HPTLC Silica Gel 60 GF_254_) by UV. The reaction mixture was concentrated under vacuum and then partitioned between ethyl acetate (40 mL) and water (5 mL). The organic layer was washed with water (10 mL x 2), brine (5 mL), dried with anhydrous sodium sulfate, and concentrated under vacuum. Flash column chromatography of the residue (silica gel, DCM/methanol = 95/5 v/v with 0.5‐1% v/v of 28–30% ammonium hydroxide) yielded compound **3e** (70.0 mg, 66.7%) as an off‐white solid. IR (KBr, cm^−1^): 3040, 1637, 1383, 1091, 751. ^1^H‐NMR (600 MHz, CDCl_3_): δ 8.39 (s, 1H), 7.94‐7.90 (m, 2H), 7.86‐7.82 (m, 2H), 7.59‐7.52 (m, 3H), 7.19 (d*, J =* 9.0 Hz, 1H), 6.82 (d, *J =* 7.8 Hz, 1H), 6.77 (s, 1H), 4.23 (t, *J =* 5.7 Hz, 2H), 3.22‐3.12 (m, 2H), 2.68 (s, 6H), 2.31‐2.24 (m, 2H). ^13^C‐NMR (150 MHz, CDCl_3_): δ 178.3, 161.1, 158.8, 158.2, 134.6, 134.0, 132.8 129.0, 128.8, 128.4, 127.9, 127.8, 127.0, 126.6, 122.3, 114.6, 110.3, 109.1, 107.7, 67.8, 56.4, 45.1, 26.4.  . HRMS (ESI) *m/z*: calcd. for C_24_H_23_NO_3_ [M+H]^+^: 374.1751, found 374.1764.

#### 4.2.8 Synthesis of 5‐(3‐(Diethylamino)Propoxy)‐2‐(Naphthalen‐2‐yl)‐4*H*‐Chromen‐4‐One (3f)

In a round‐bottomed flask, compound **2** (167 mg, 0.408 mmol) was added in 8 mL of DMF and gently heated until it was fully dissolved followed by addition of potassium carbonate (0.168 g, 1.13 mmol, 3 equiv.) and diethylamine (253 µL, 2.45 mmol, 6 equiv.). The reaction mixture was stirred at room temperature for 21 h. The reaction was monitored using TLC (R_f_ = 0.71, DCM/methanol = 90:10 v/v, HPTLC Silica Gel 60 GF_254_) by UV. The reaction mixture was concentrated under vacuum and then partitioned between ethyl acetate (40 mL) and water (5 mL). The organic layer was washed with water (5 mL), brine (5 mL), dried with anhydrous sodium sulfate, and concentrated under vacuum. Flash column chromatography of the residue (silica gel, DCM/methanol = 95/5 v/v with 0.5‐1% v/v of 28–30% ammonium hydroxide) yielded compound **3f** (152 mg, 92.8%) as an off‐white solid. IR (KBr, cm^−1^): 2966, 1603, 1460, 1384, 1091, 748. ^1^H‐NMR (600 MHz, CDCl_3_): δ 8.36 (s, 1H), 7.91‐7.87 (m, 2H), 7.83‐7.79 (m, 2H), 7.55‐7.60 (m, 3H), 7.15 (d, *J* = 8.4 Hz, 1H), 6.78 (d, *J* = 8.4 Hz, 1H), 6.75 (s, 1H), 4.17 (t, *J* = 6.0 Hz, 2H), 3.13‐3.03 (m, 2H), 2.86 (q, *J* = 7.2 Hz, 4H), 2.22 (quint, *J* = 6.0, 2H), 1.20 (t, *J* = 7.2 Hz, 6H). ^13^C‐NMR (150 MHz, CDCl_3_): δ 178.3, 161.3, 158.8, 158.3, 134.6, 134.0, 132.9, 129.1, 128.9, 128.5, 128.0, 127.9, 127.1, 126.7, 122.4, 114.6, 110.4, 109.3, 107.7, 67.6, 49.7, 47.3, 25.7, 10.6. HRMS (ESI) *m/z*: calcd. for C_26_H_27_NO_3_ [M+H]^+^: 402.2064, found 402.2081.

#### 4.2.9 Synthesis of 5‐(3‐(Dipropylamino)Propoxy)‐2‐(Naphthalen‐2‐yl)‐4*H*‐Chromen‐4‐One (3g)

In a round‐bottomed flask, compound **2** (100 mg, 0.244 mmol) was added in 8 mL of DMF and gently heated until it was fully dissolved followed by addition of potassium carbonate (0.101 g, 0.731 mmol, 3 equiv.) and dipropylamine (201 µL, 1.47 mmol, 6 equiv.). The reaction mixture was stirred at room temperature for 21 h. The reaction was monitored using TLC (R_f_ = 0.71, DCM/methanol = 90:10 v/v, HPTLC Silica Gel 60 GF_254_) by UV. The reaction mixture was concentrated under vacuum and then partitioned between ethyl acetate (40 mL) and water (5 mL). The organic layer was washed with water (10 mL x 2), brine (5 mL), dried with anhydrous sodium sulfate, and concentrated under vacuum. Flash column chromatography of the residue (silica gel, DCM/methanol = 95/5 v/v with 0.5‐1% v/v of 28–30% ammonium hydroxide) yielded compound **3** **g** (88.0 mg, 84.0%) as a light‐brown solid. IR (KBr, cm^−1^): 2964, 2455, 1640, 1602, 1460, 1265, 1091, 749. ^1^H‐NMR (600 MHz, CDCl_3_): δ 8.38 (s, 1H), 7.92‐7.89 (m, 2H), 7.84‐7.80 (m, 2H), 7.58 (t, *J* = 8.1 Hz, 1H), 7.55‐7.51 (m, 2H), 7.20 (d, *J* = 8.4 Hz, 1H), 6.80 (d, *J* = 8.4 Hz, 1H), 6.74 (s, 1H), 4.24 (t, *J* = 5.1 Hz, 2H), 3.57 (t, *J* = 6.9 Hz, 2H), 3.14‐3.05 (m, 4H), 2.51‐2.43 (m, 2H), 1.93‐1.83 (m, 4H), 0.97 (t, *J* = 7.5 Hz, 6H). ^13^C‐NMR (150 MHz, CDCl_3_): δ 178.5, 161.8, 158.2, 158.0, 134.7, 134.4, 132.9, 129.1, 129.0, 128.3, 128.2, 127.9, 127.2, 126.8, 122.3, 114.4, 111.0, 109.1, 107.8, 67.1, 55.0, 51.6, 24.3, 16.9, 11.3. HRMS (ESI) *m/z*: calcd. for C_28_H_31_NO_3_ [M+H]^+^: 430.2377, found 430.2397.

#### 4.2.10 Synthesis of 5‐Hydroxy‐2‐(Naphthalen‐1‐yl)‐4*H*‐Chromen‐4‐One (6)

In a round‐bottomed flask, 2′, 6′‐dihydroxyacetophenone (0.250 g, 1.64 mmol) was dissolved in 10 mL of acetone followed by addition of potassium carbonate (1.14 g, 8.21 mmol, 5 equiv.) and the reaction mixture was stirred at room temperature for 10 min. After addition of 1‐naphthoyl chloride (248 µL, 1 equiv.), the reaction mixture was refluxed at 75°C for 3 h. The reaction was monitored using TLC (R_f_ = 0.34, hexane/ethyl acetate = 95:5 v/v, HPTLC Silica Gel 60 GF_254_) by UV. The reaction mixture was concentrated under vacuum and then partitioned between ethyl acetate (50 mL) and water (5 mL). The organic layer was further washed with water (5 mL) and brine (5 mL), dried with anhydrous sodium sulfate, and concentrated under vacuum. Flash column chromatography of the residue (silica gel, hexane/ethyl acetate = 95:5 v/v) yielded compound **6** (107 mg, 22.7%) as a white solid. IR (KBr, cm^−1^): 2923, 1654, 1413, 1259, 791, 751.^1^H‐NMR (600 MHz, CDCl_3_): δ 8.13‐8.12 (m, 1H), 8.04 (d, *J* = 8.4 Hz, 1H), 7.97‐7.95 (m, 1H), 7.76 (d, *J* = 6.0 Hz, 1H), 7.61‐7.56 (m, 4H), 6.98 (d, *J* = 7.8 Hz, 1H), 6.88 (d, *J* = 8.4, 1H), 6.63 (s, 1H), 1.60 (broad, 1H).^13^C‐NMR (150 MHz, CDCl_3_): δ 183.6, 166.8, 161.0, 157.0, 135.6, 133.8, 132.0, 130.4, 130.2, 128.9, 128.2, 127.7, 126.8, 125.1, 124.8, 111.7, 111.6, 111.0, 107.3. HRMS (ESI) *m/z*: calcd. for C_19_H_12_O_3_ [M+H]^+^: 289.0859, found 289.0844.

#### 4.2.11 Synthesis of 5‐(3‐Bromopropoxy)‐2‐(Naphthalen‐1‐yl)‐4*H*‐Chromen‐4‐One (7)

In a small round‐bottomed flask, *compound* ***6*** (135 mg, 0.468 mmol) was dissolved in 5 mL of DMF at 80°C and cooled to room temperature followed by addition of potassium carbonate (0.194 g, 1.40 mmol, 3 equiv.). The reaction mixture was stirred at room temperature for 10 min. After addition of 1,3‐dibromobutane (286 µL, 2.81 mmol, 6 equiv.), the reaction mixture was stirred at room temperature for 4 days. The reaction was monitored using TLC (R_f_ = 0.23, hexane/ethyl acetate = 85:15 v/v, HPTLC Silica Gel 60 GF_254_) by UV. The reaction mixture was concentrated under vacuum and then partitioned between ethyl acetate (50 mL) and water (10 mL). The organic layer was then washed with water (5 mL x 2), brine (5 mL), dried with anhydrous sodium sulfate, and concentrated under vacuum. Flash column chromatography of the residue (silica gel, hexane/ethyl acetate = 75:25 v/v) yielded compound **7** (130 mg, 67.9%) as a white solid. IR (KBr, cm^−1^): 1643, 1599, 1475, 1454, 1371, 1307, 1072, 763. ^1^H‐NMR (600 MHz, CDCl_3_): δ 8.14‐8.11 (m, 1H), 7.99 (d, *J* = 6.0 Hz, 1H), 7.93‐7.90 (m, 1H), 7.73 (dd, *J* = 1.2, 7.2 Hz, 1H), 7.56‐7.53 (m, 4H), 7.07 (dd, *J* = 0.6, 8.4 Hz, 1H), 6.86 (d, *J* = 7.8 Hz, 1H), 6.54 (s, 1H), 4.27 (t, *J* = 5.4 Hz, 2H), 3.89 (t, *J* = 6.3 Hz, 2H), 2.45 (quint, *J* = 5.8 Hz, 2H).^13^C‐NMR (150 MHz, CDCl_3_): δ 178.0, 163.2, 159.2, 158.8, 133.9, 133.8, 131.5, 130.5, 130.4, 128.8, 127.9, 127.5, 126.6, 125.2, 125.0, 115.0, 114.7, 110.6, 108.0, 66.6, 32.2, 30.9. HRMS (MALDI) *m/z*: calcd. for C_22_H_17_BrO_3_ [M+H]^+^: 409.0434, found 409.0230.

#### 4.2.12 Synthesis of 2‐(Naphthalen‐1‐yl)‐5‐(3‐(Piperidin‐1‐yl)Propoxy)‐4*H*‐Chromen‐4‐One (4b)

In a round‐bottomed flask, compound **7** (140 mg, 0.342 mmol) was dissolved in 8 mL of DMF followed by addition of potassium carbonate (0.142 g, 1.03 mmol, 3 equiv.) and piperidine (203 µL, 2.05 mmol, 6 equiv.). The reaction mixture was stirred at room temperature for 2 days. The reaction was monitored using TLC (R_f_ = 0.29, DCM/methanol = 95:5 v/v, HPTLC Silica Gel 60 GF_254_) by UV. The reaction mixture was concentrated under vacuum and then partitioned between ethyl acetate (50 mL) and water (5 mL). The organic layer was further washed with brine (5 mL), dried with anhydrous sodium sulfate, and concentrated under vacuum. Flash column chromatography of the residue (silica gel, DCM/methanol = 95:5 v/v followed by DCM/methanol/28‐30% ammonium hydroxide (95/5/1, v/v) yielded compound **4b** (124 mg, 87.7%) as an off‐white solid. IR (KBr, cm^−1^): 2500, 1645, 1603, 1459, 1375, 1274, 1087, 779. ^1^H‐NMR (600 MHz, CDCl_3_): δ 8.10‐8.08 (m, 1H), 8.01 (d, *J* = 7.8 Hz, 1H), 7.95‐7.92 (m, 1H), 7.73 (dd, *J* = 1.2, 7.2 Hz, 1H), 7.61‐7.54 (m, 4H), 7.11 (dd, *J* = 0.6, 8.4 Hz, 1H), 6.85 (d, *J* = 7.8 Hz, 1H), 6.52 (s, 1H), 4.26 (t, *J* = 5.4 Hz, 2H), 3.52 (t, *J* = 7.2 Hz, 2H), 3.48‐2.82 (m, 4H), 2.55 (quint, 6.0 Hz, 2H), 2.24‐1.89 (m, 4H), 1.80‐1.50 (m, 2H). ^13^C‐NMR (150 MHz, CDCl_3_): δ 178.3, 163.9, 158.7, 158.4, 134.4, 133.8, 131.7, 130.4, 130.1, 128.9, 128.0, 127.6, 126.7, 125.1, 124.8, 114.6, 114.4, 111.1, 108.1, 67.0, 55.8, 53.8, 24.0, 22.7, 22.2. HRMS (MALDI) *m/z*: calcd. for C_27_H_27_NO_3_ [M+H]^+^: 414.2064, found 414.2001.

### 4.3 Preparation of DNA Samples

#### 4.3.1 Preparation of Poly(dA)•2poly(dT) for Thermal Denaturation

A solution (1 mL) of poly(dA)•poly(dT) (15 µM/base pair) and poly(dT) (15 µM/base) in sodium cadodylate buffer (10 mM, pH 7.0) and KCl (150 mM) in the presence and absence of a ligand of interest (**1**, **3a**‐**3g**, **6**, **5b**, and **4b**) at a desired concentration was heated at 95°C for 5 min, slowly cooled to 25°C, and incubated at 4°C overnight.

#### 4.3.2 Preparation of the Intramolecular Oligonucleotide Triplex (DNA1) for Thermal Denaturation

A solution (1 mL) of **DNA1** (1 µM/strand) in sodium cadodylate buffer (10 mM, pH 7.0) and NaCl (100 mM) in the presence and absence of a ligand of interest (**1**, **3a**‐**3g**, **6**, **5b**, and **4b**) at a desired concentration was heated at 95°C for 5 min, slowly cooled to 25°C, and incubated at 4°C overnight.

#### 4.3.3 Preparation of DNA1, DNA2, and DNA4 for Circular Dichroism

A solution (2 mL) of **DNA1**, **DNA2**, or **DNA4** (5 µM/strand) in sodium cacodylate buffer (10 mM, pH 7.0) and NaCl (100 mM) was heated at 95°C for 5 min, slowly cooled to 25°C, and incubated at 4°C overnight.

### 4.4 UV Thermal Denaturation of Triplex DNA

The UV melting spectra of DNA triplex samples (1 mL) were recorded in 1 cm path‐length quartz cuvettes at 260 nm, 280 nm, and 284 nm as a function of temperature (25‐90°C, heating rate: 0.2°C/min). Melting temperatures (T_m_s) were determined using the first derivative method. All experiments were carried out in duplicate.

### 4.5 Circular Dichroism Titration Measurements

Aliquots of **3b** (in DMSO) were gradually titrated into a solution (2 mL) of **DNA1** (5 µM), **DNA2** (5 µM), or **DNA4** (5 µM) in sodium cacodylate (10 mM, pH 7.0) and 100 mM NaCl at 15°C. The final concentrations of **4b** were 0, 1, 2.5, 5, 10, 15, and 20 µM. The % DMSO in solution was kept at no more than 1%. After each addition, the solution was mixed by gently swirling the close‐capped cuvette. The interval time between each addition was 5 min for equilibrium. CD spectra were recorded from 500 to 230 nm with a 100 nm/min scan speed.

### 4.6 Fluorescent Intercalator Displacement Assay

Thiazole orange (3 µM) was first incubated with a solution of **DNA1** (1.5 µM) in sodium cacodylate (10 mM, pH 7.0) and 100 mM NaCl for 15 min in the dark. Aliquots of **3b**, **3** **g**, or **1** were titrated into the reaction mixture and equilibrated for 5 min in the dark before measurement. The fluorescence spectra (Ex: 509 nm, slit width: 2.5 nm, scan speed 200 nm/min) were recorded from 515 to 700 nm after each addition. The percent TO fluorescence (%TO) was calculated at lambda max using the equation %TO = (FA/FA_0_) × 100, where FA_0_ is the initial fluorescence intensity in the absence of a ligand, and FA is the fluorescence intensity with ligand addition.

### 4.7 Viscosity Experiments

The poly(dA)•2poly(dT) sample (2 mL) was prepared by mixing poly(dA)•poly(dT) (0.5 mM/base triplet) and poly(dT) (0.5 mM/base triplet) in a sodium cacodylate buffer (10 mM) and KCl (150 mM) at pH 7.0. The DNA solution was heated at 95°C for 5 min, slowly cooled to 25°C, and incubated at 4°C overnight to form the corresponding triplex. Calf thymus DNA (1 mM, 2 mL) was prepared in a sodium cacodylate buffer (10 mM) and KCl (150 mM) at pH 7.0. Small aliquots of a concentrated stock solution of the desired ligand (**3b** for triplex and **EB** for duplex) were added to the poly(dA)•2poly(dT) sample (0.5 mM) or calf thymus DNA (1 mM) in an Ostwald‐type viscometer at room temperature to obtain the desired ligand/DNA ratios. After each addition of ligand, the flow time for the level of the solution to pass between the two marks on the viscometer was recorded using a stopwatch. The measurements were triplicated for each ligand/DNA ratio. The relative viscosities were calculated based on published equations [34].

### 4.8 Visualization of Triplex Formation by Native PAGE

The triplex samples for native PAGE were prepared by mixing **DNA5** (3 pmol), **DNA6** (3 pmol), and **TFO1** (3 pmol) in 20 µL of Tris‐acetate buffer (90 mM, pH 5.0) in the absence and presence of ligand **3b** (0.1, 0.5, 1, 2.5, 5, 10, and 20 µM). The duplex sample for native PAGE was prepared by mixing **DNA5** (3 pmol) and **DNA6** (3 pmol) in 20 µL of Tris‐acetate buffer (90 mM, pH 5.0). The DNA (triplex and duplex) solutions were heated to 95°C for 5 min, slowly cooled down to room temperature, and stored at 4°C overnight. Samples of **DNA5** (3 pmol) only, **DNA6** (3 pmol) only, and **TFO1** (3 pmol) only were also made as control samples. DNA separations were carried out using a 12% nondenaturing polyacrylamide gel made in a Tris‐acetate buffer (90 mM, pH 5.0). DNA bands were visualized using the corresponding fluorescence channels for Cy3 and Cy5 on an Invitrogen iBright FL1500 imaging system and quantified by iBright analysis software.

### 4.9 Fluorescence Induction by Binding of 3b With Triplex DNA Under UV Illumination

Solutions (2 mL) of **DNA1** or **DNA4** (1 µM/strand) in sodium cacodylate buffer (10 mM, pH 7.0) and NaCl (100 mM) in the presence and absence of a ligand of interest (**5, 5b, 1,** and **3b**) at 10 µM were prepared in microcentrifuge tubes. The solutions were heated at 95°C for 5 min, slowly cooled to 25°C, and incubated at 4°C overnight. The solutions were transferred to glass vials and exposed to 365 nm UV light using a handheld UV lamp in the dark.

The solutions used above were then diluted fivefold using the same buffer to a final volume of 2 mL. The fluorescence spectra (Ex: 323 nm, slit width: 5.0 nm, scan speed 300 nm/min) of the final solutions were recorded from 340–625 nm at 15°C.

### 4.10 Inhibition of DraI Activity by Ligand‐mediated Triplex Formation

Samples were prepared by mixing 300 ng of plasmid, **TFO2** (10 µM), *DraI* (20 units), and NaCl (50 mM) in the rCutSmart Buffer (New England Biolabs, USA) in the absence and presence of ligand **3b** (2.5, 5, 10, 15, and 20 µM). Control samples of the plasmid without *DraI*, **DNA7** with *DraI* only, and **DNA7** and **TFO2** with *DraI* were also prepared. Samples were incubated in a 37°C water bath for 2 h before they were incubated at 65°C for 20 min to inactivate *DraI*. The resulting DNA was resolved on a 1% agarose gel and visualized by staining with ethidium bromide using an Invitrogen iBright FL1500 imaging system. Quantification of DNA fragments was carried out using Image Lab analysis software.

### 4.11 Statistical Analysis

Statistical analysis was performed using GraphPad Prism 10 software. For the UV thermal denaturation experiments (n = 2 per group), a two‐tailed unpaired t‐test was conducted to assess differences between groups. Results were presented as statistically significant (P < 0.05), very significant (P < 0.01 or 0.001), and insignificant (P > 0.05).

## Conflicts of Interest

The authors declare that they have no known competing financial interests or personal relationships that could have appeared to influence the work reported in this paper.

## Supporting information




**Supporting File 1**: chem70673‐sup‐0001‐SuppMat.pdf.

## Appendix A Supplementary data

### A.1

The sequence of **DNA7** and ^1^H‐ and ^13^C‐NMR spectra of all compounds.
