# Supplementary material for: Highly Potent Fluorogenic Ligands for Triplex DNA: 5‐Substituted 2‐(Naphthalen‐2‐yl)‐4H‐Chromen‐4‐Ones
Source: Chemistry. 2026 Jan 12;32(30):e03318. doi: 10.1002/chem.202503318 (PMC13472347; doi:10.1002/chem.202503318)
Supplement: Supplementary file 1 [file CHEM-32-e03318-s001.pdf]

Highly Potent Fluorogenic Ligands for Triplex DNA: 5-Substituted 2-(Naphthalen-2-yl)-4*H*-chromen-4-ones

Nghia Tran, Landy Gu, Krege, M. Christison, Kalee Thao, and Liang Xue*

Department of Chemistry, University of the Pacific, 3601 Pacific Avenue, Stockton, California 95211 (USA)

Table of Contents:

Supporting Information Figure S1. The entire sequence of the constructed plasmid (**DNA7**).

Supporting Information Figure S2. ^{1}H NMR of **1**.

Supporting Information Figure S3. ^{13}C NMR of **1**.

Supporting Information Figure S4. ^{1}H NMR of **2**.

Supporting Information Figure S5. ^{13}C NMR of **2**.

Supporting Information Figure S6. ^{1}H NMR of **3a**.

Supporting Information Figure S7. ^{13}C NMR of **3a**.

Supporting Information Figure S8. ^{1}H NMR of **3b**.

Supporting Information Figure S9. ^{13}C NMR of **3b**.

Supporting Information Figure S10. ^{1}H NMR of **3c**.

Supporting Information Figure S11. ^{13}C NMR of **3c**.

Supporting Information Figure S12. ^{1}H NMR of **3d**.

Supporting Information Figure S13. ^{13}C NMR of **3d**.

Supporting Information Figure S14. ^{1}H NMR of **3e**.

Supporting Information Figure S15. ^{13}C NMR of **3e**.

Supporting Information Figure S16. ^{1}H NMR of **3f**.

Supporting Information Figure S17. ^{13}C NMR of **3f**.

Supporting Information Figure S18. ^{1}H NMR of **3g**.

Supporting Information Figure S19. ^{13}C NMR of **3g**.

Supporting Information Figure S20. ^{1}H NMR of **6**.

Supporting Information Figure S21. ^{13}C NMR of **6**.

Supporting Information Figure S22. ^{1}H NMR of **7**.

Supporting Information Figure S23. ^{13}C NMR of **7**.

Supporting Information Figure S24. ^{1}H NMR of **4b**.

Supporting Information Figure S25. ^{13}C NMR of **4b**.

CAGTCACGACGTTGTAAAACGACGGCCAGTGAGCGCGTAATACGACTCACTATAGGGCGAATTGGGTACCTTTAA
AAAAAAAAAAAAAAATGGTATCAAAAGGAGAGGAAGATAATATGGCGATTATTAAAGAATTCATGCGTTTTAAGGTGC
ATATGGAAGGTAGTGTTAATGGTCACGAATTCGAGATCGAGGGTGAGGGCGAGGGTCGTCCGTATGAAGGCACTCA
AACCGCAAAATTGAAGGTAACGAAAGGCGGACCGCTCCCTTTCGCTTGGGATATCCTGTCCCCGCAGTTTATGTACG
GCAGCAAAGCGTATGTCAAACACCCGGCTGATATCCCGGATTACCTGAAGTTGTCGTTCCCGGAAGGTTTTAAGTGG
GAGCGCGTGATGAACTTTGAGGACGGTGGCGTCGTGACGGTGACCCAAGACAGCTCCCTGCAGGACGGCGAGTTC
ATCTACAAGGTGAAACTGCGTGGTACGAACTTCCCAAGCGATGGCCCGGTTATGCAGAAAAAGACCATGGGTTGGG
AGGCCAGCTCTGAACGCATGTACCCGGAGGACGGCGCGCTGAAAGGTGAAATCAAGCAGCGTTTGAAGTTAAAGG
ACGGCGGCCACTACGACGCGGAAGTTAAAACCACCTATAAGGCCAAAAAGCCGGTTCAGCTGCCGGGTGCGTACAA
CGTGAACATTAAGCTGGATATTACCAGCCATAATGAAGACTACACCATTGTTGAACAATATGAGCGCGCAGAGGGTCG
TCATAGCACCGGTGGTATGGATGAACTGTATAAAGGTACCGGGCCCCCCCTCGAGGTCGACGGTATCGATAAGCTTG
ATATCGAATTCCTGCAGCCCGGGGGATCCACTAGTTCTAGAGCGGCCGCCACCGCGGTGGAGCTCCAGCTTTTGTTC
CCTTTAGTGAGGGTTAATTGCGCGCTTGGCGTAATCATGGTCATAGCTGTTTCCTGTGTGAAATTGTTATCCGCTCACA
ATTCCACACAACATACGAGCCGGAAGCATAAAGTGTAAAGCCTGGGGTGCCTAATGAGTGAGCTAACTCACATTAATT
GCGTTGCGCTCACTGCCCGCTTTCCAGTCGGGAAACCTGTCGTGCCAGCTGCATTAATGAATCGGCCAACGCGCGGG
GAGAGGCGGTTTGCGTATTGGGCGCTCTTCCGCTTCCTCGCTCACTGACTCGCTGCGCTCGGTCGTTCGGCTGCGGC
GAGCGGTATCAGCTCACTCAAAGGCGGTAATACGGTTATCCACAGAATCAGGGGATAACGCAGGAAAGAACATGTG
AGCAAAAGGCCAGCAAAAGGCCAGGAACCGTAAAAAGGCCGCGTTGCTGGCGTTTTTCCATAGGCTCCGCCCCCCT
GACGAGCATCACAAAAATCGACGCTCAAGTCAGAGGTGGCGAAACCCGACAGGACTATAAAGATACCAGGCGTTTC
CCCCTGGAAGCTCCCTCGTGCGCTCTCCTGTTCCGACCCTGCCGCTTACCGGATACCTGTCCGCCTTTCTCCCTTCGG
GAAGCGTGGCGCTTTCTCATAGCTCACGCTGTAGGTATCTCAGTTCGGTGTAGGTCGTTCGCTCCAAGCTGGGCTGT
GTGCACGAACCCCCCGTTCAGCCCGACCGCTGCGCCTTATCCGGTAACTATCGTCTTGAGTCCAACCCGGTAAGACAC
GACTTATCGCCACTGGCAGCAGCCACTGGTAACAGGATTAGCAGAGCGAGGTATGTAGGCGGTGCTACAGAGTTCTT
GAAGTGGTGGCCTAACTACGGCTACACTAGAAGGACAGTATTTGGTATCTGCGCTCTGCTGAAGCCAGTTACCTTCG
GAAAAAGAGTTGGTAGCTCTTGATCCGGCAAACAAACCACCGCTGGTAGCGGTGGTTTTTTTGTTTGCAAGCAGCA
GATTACGCGCAGAAAAAAAGGATCTCAAGAAGATCCTTTGATCTTTTCTACGGGGTCTGACGCTCAGTGGAACGAAA
ACTCACGTTAAGGGATTTTGGTCATGAGATTATCAAAAAGGATCTTCACCTAGATCCTTTTAAATTAAAAATGAAGTTT
TAAATCAATCTAAAGTATATATGAGTAAACTTGGTCTGACAGTTACCAATGCTTAATCAGTGAGGCACCTATCTCAGCG
ATCTGTCTATTTCGTTCATCCATAGTTGCCTGACTCCCCGTCGTGTAGATAACTACGATACGGGAGGGCTTACCATCTG
GCCCCAGTGCTGCAATGATACCGCGAGACCCACGCTCACCGGCTCCAGATTTATCAGCAATAAACCAGCCAGCCGGA
AGGGCCGAGCGCAGAAGTGGTCCTGCAACTTTATCCGCCTCCATCCAGTCTATTAATTGTTGCCGGGAAGCTAGAGT
AAGTAGTTCGCCAGTTAATAGTTTGCGCAACGTTGTTGCCATTGCTACAGGCATCGTGGTGTCACGCTCGTCGTTTGG
TATGGCTTCATTCAGCTCCGGTTCCCAACGATCAAGGCGAGTTACATGATCCCCCATGTTGTGCAAAAAAGCGGTTAG
CTCCTTCGGTCCTCCGATCGTTGTCAGAAGTAAGTTGGCCGCAGTGTTATCACTCATGGTTATGGCAGCACTGCATAAT
TCTCTTACTGTCATGCCATCCGTAAGATGCTTTTCTGTGACTGGTGAGTACTCAACCAAGTCATTCTGAGAATAGTGTAT
GCGGCGACCGAGTTGCTCTTGCCCGGCGTCAATACGGGATAATACCGCGCCACATAGCAGAACTTTAAAAGTGCTCA
TCATTGGAAAACGTTCTTCGGGGCGAAAACTCTCAAGGATCTTACCGCTGTTGAGATCCAGTTCGATGTAACCCACTC
GTGCACCCAACTGATCTTCAGCATCTTTTACTTTCACCAGCGTTTCTGGGTGAGCAAAAACAGGAAGGCAAAATGCC
GCAAAAAAGGGAATAAGGGCGACACGGAAATGTTGAATACTCATACTCTTCCTTTTTCAATATTATTGAAGCATTTATC
AGGGTTATTGTCTCATGAGCGGATACATATTTGAATGTATTTAGAAAAATAAACAAATAGGGGTTCCGCGCACATTTCC
CCGAAAAGTGCCAC-3’

Supporting Information Figure S1. The entire sequence of the constructed plasmid (**DNA7**).

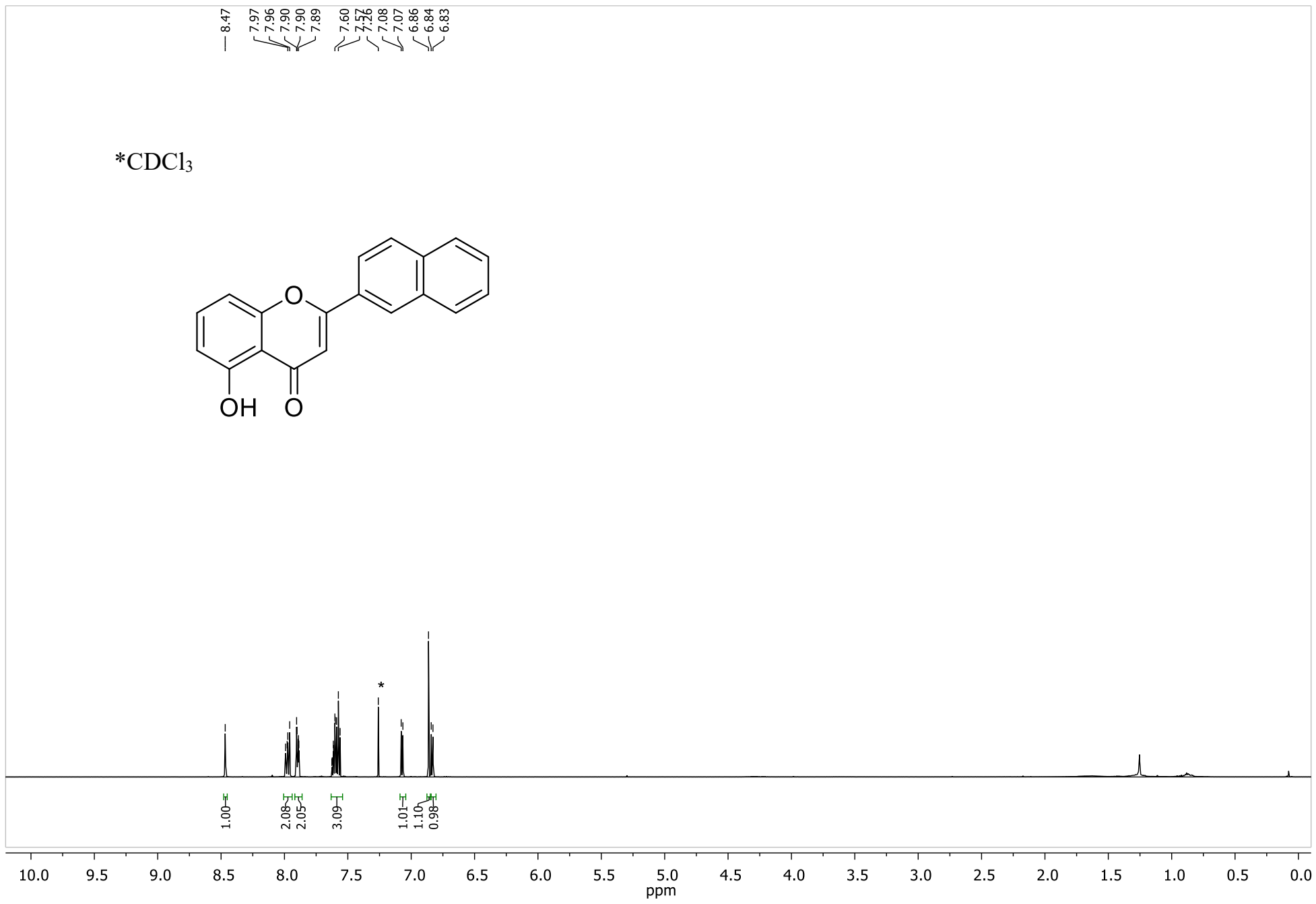

Supporting Information Figure S2. ^{1}H NMR of **1**.

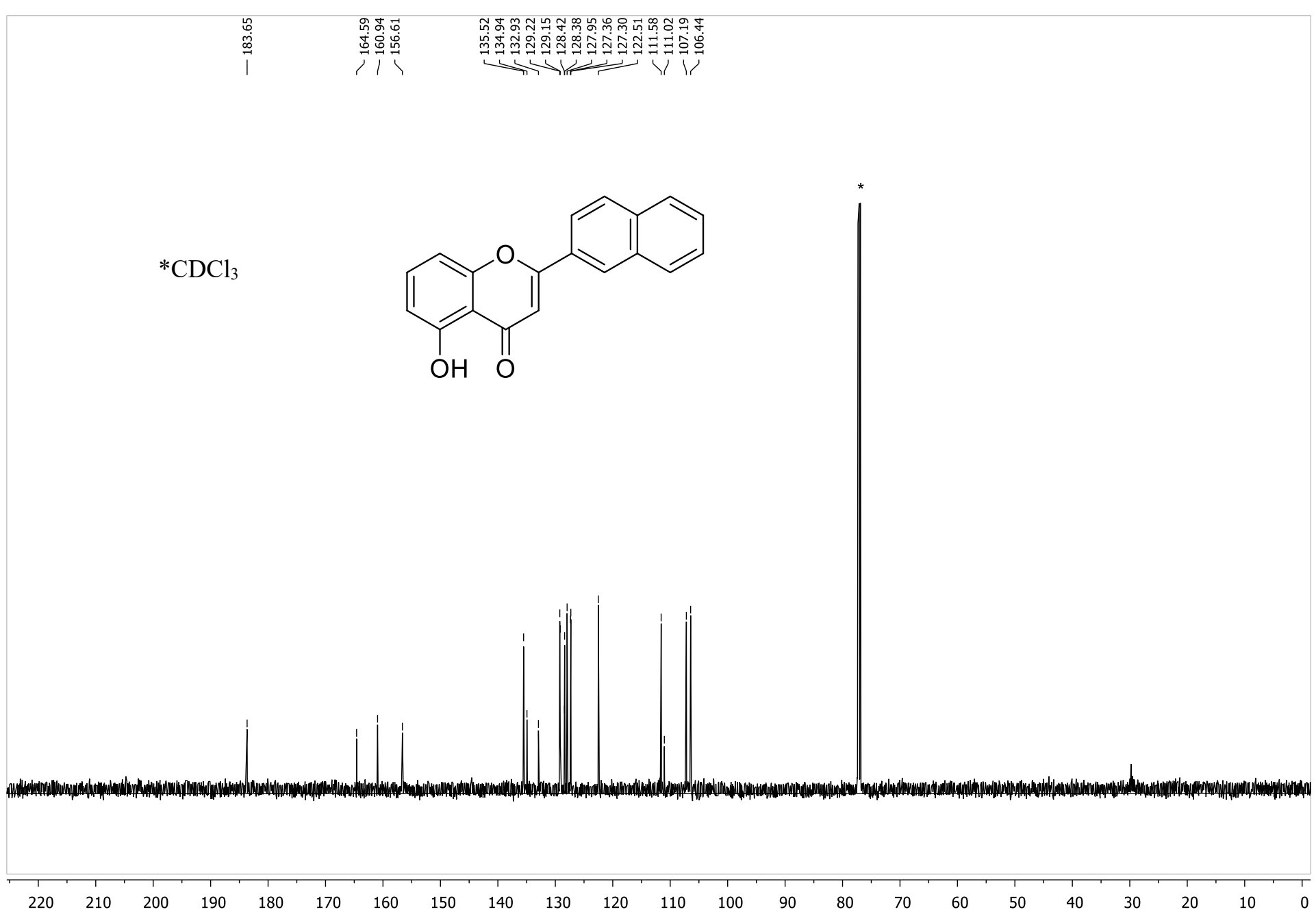

Supporting Information Figure S3. ^{13}C NMR of **1**.

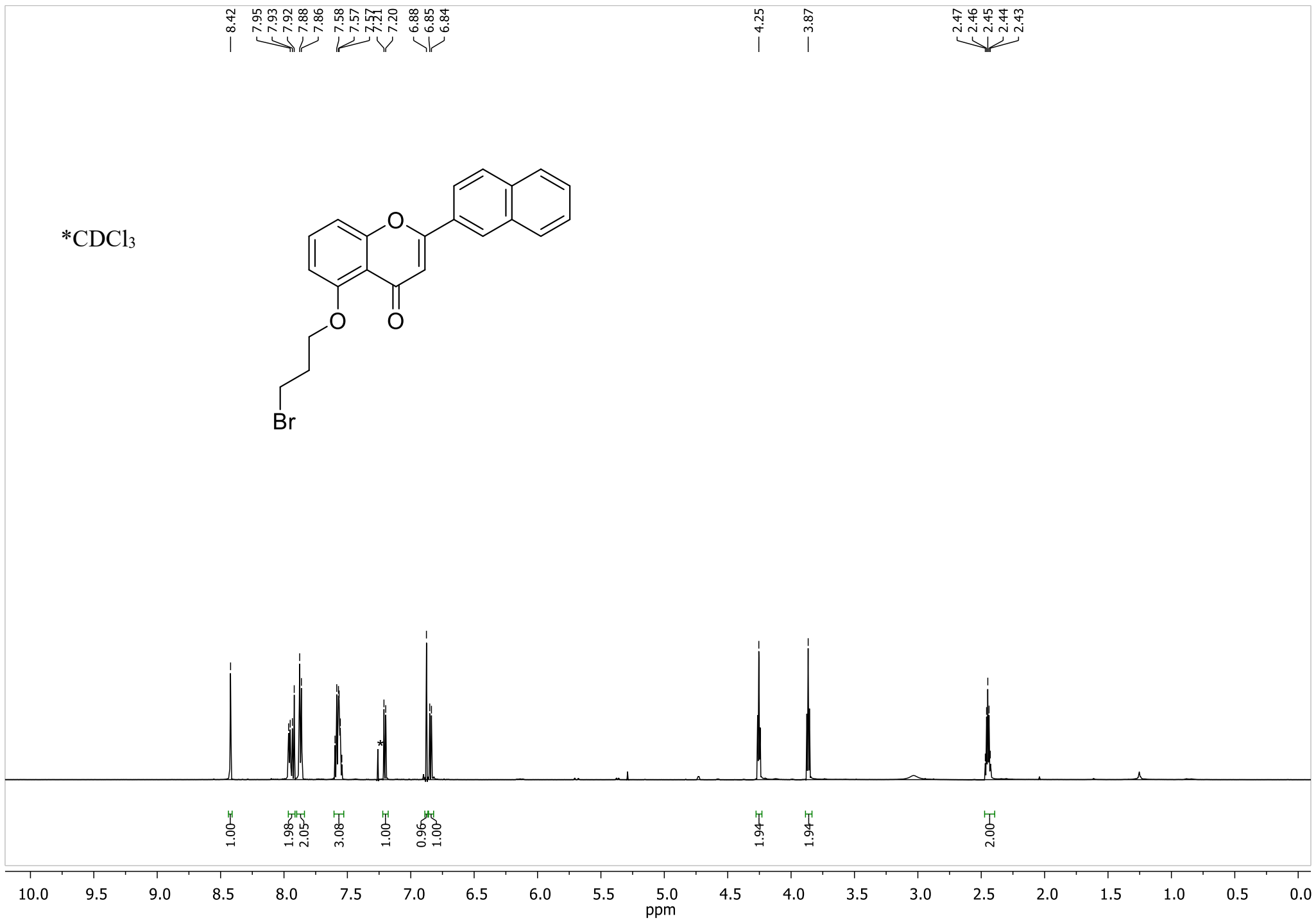

Supporting Information Figure S4. ^{1}H NMR of **2**.

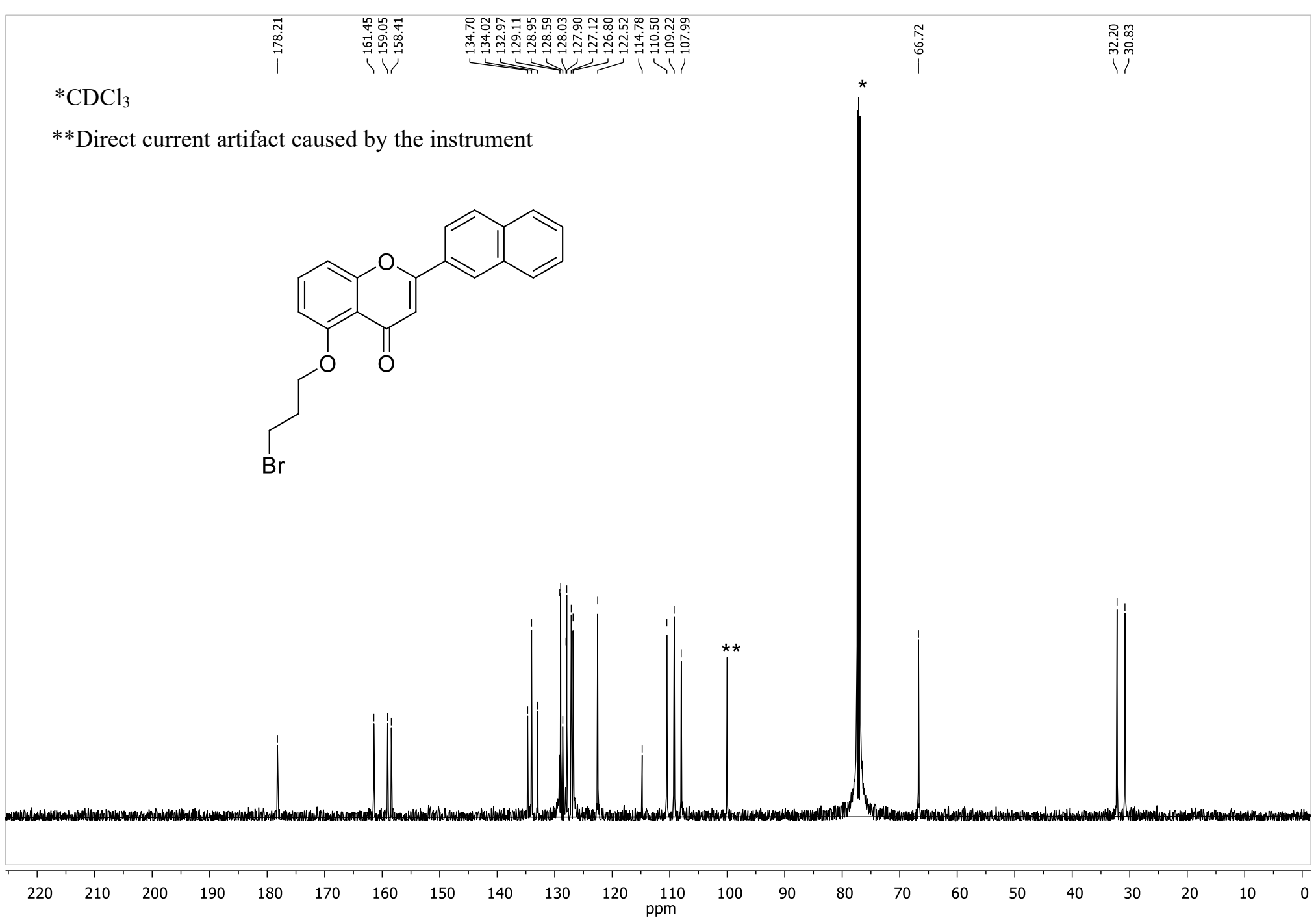

Supporting Information Figure S5. ^{13}C NMR of **2**.

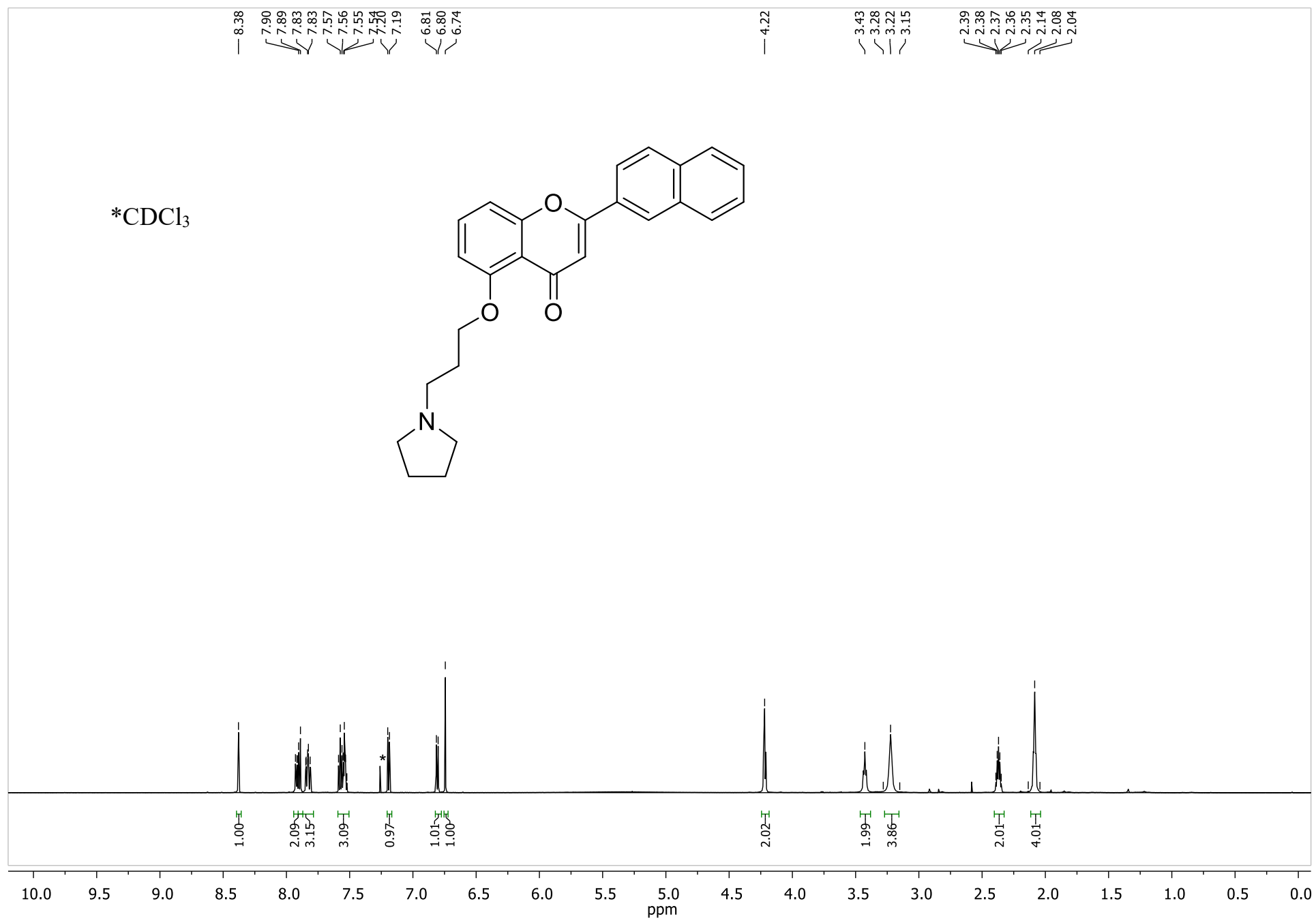

Supporting Information Figure S6. ^{1}H NMR of **3a**.

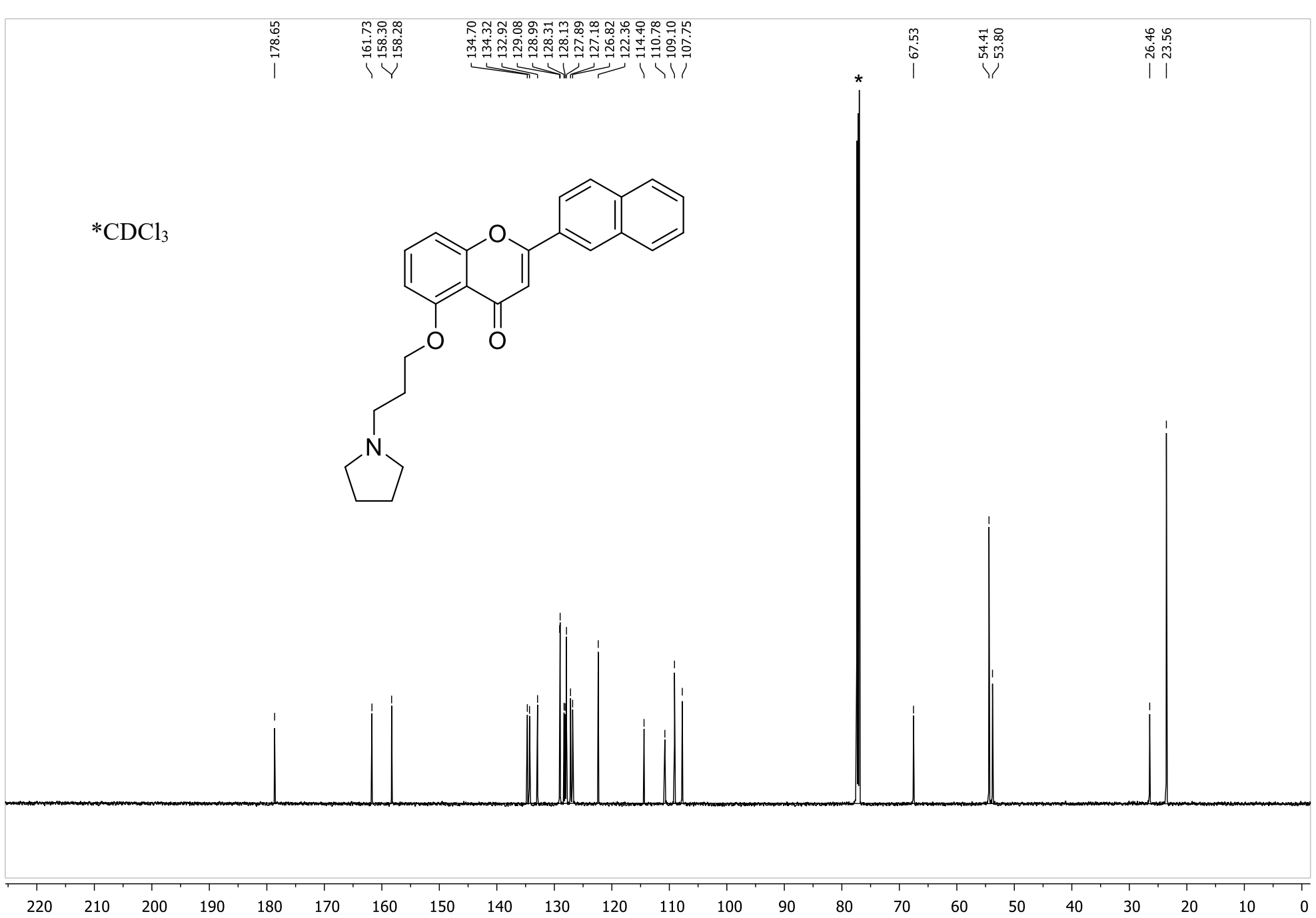

Supporting Information Figure S7. ^{13}C NMR of **3a**.

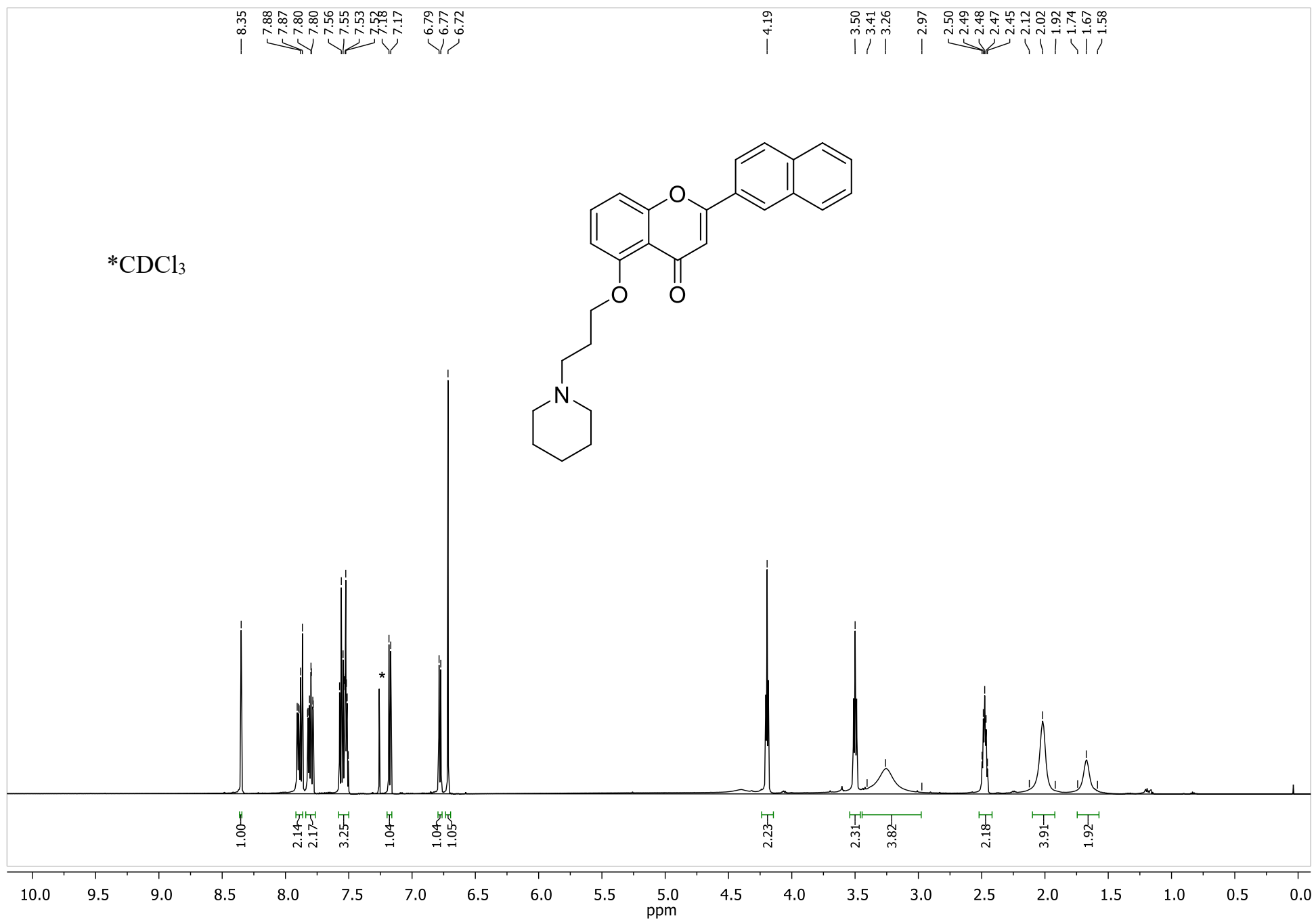

Supporting Information Figure S8. ^{1}H NMR of **3b**.

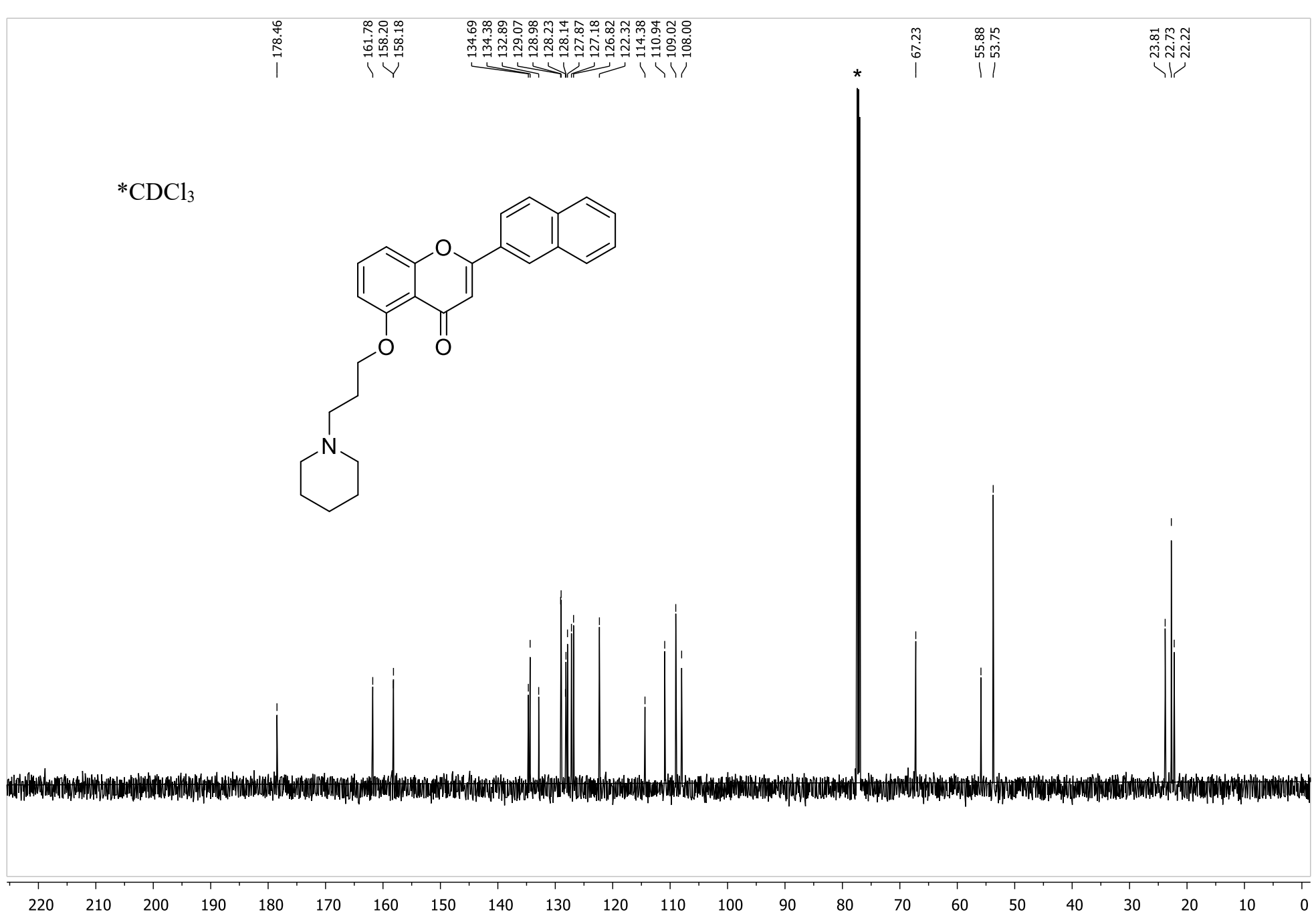

Supporting Information Figure S9. ^{13}C NMR of **3b**.

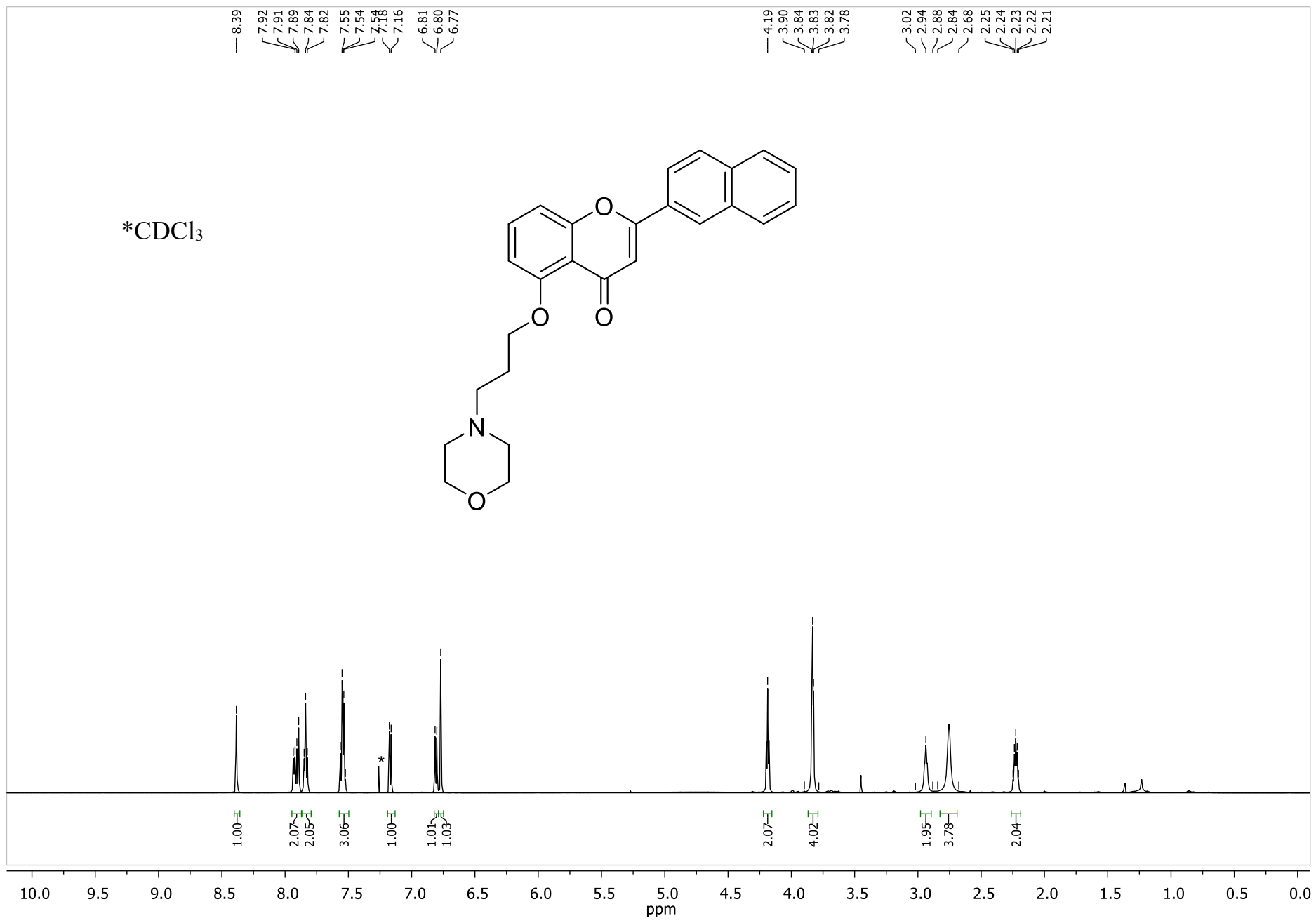

Supporting Information Figure S10. ^{1}H NMR of **3c**.

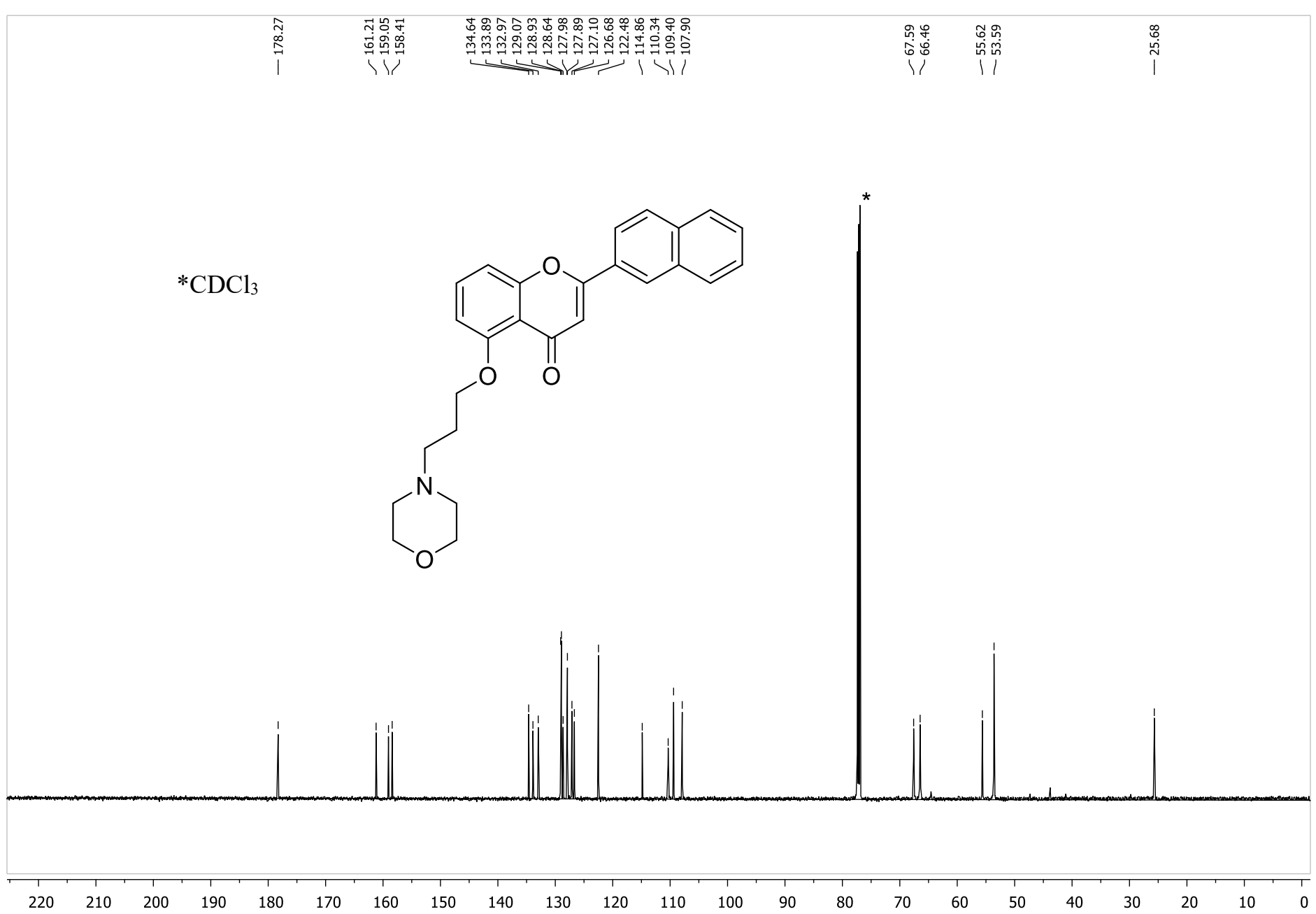

Supporting Information Figure S11. ^{13}C NMR of **3c**.

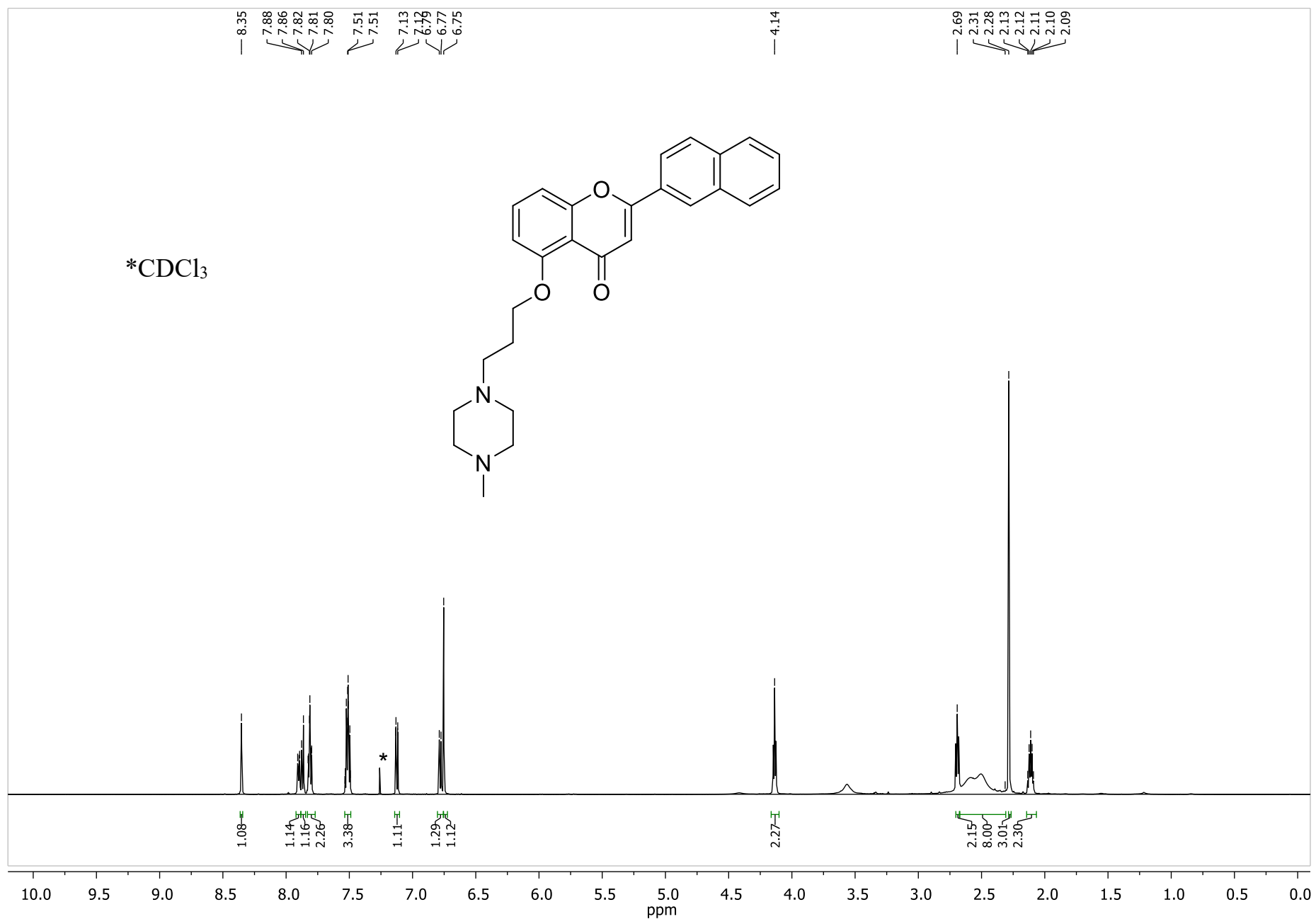

Supporting Information Figure S12. ^{1}H NMR of **3d**.

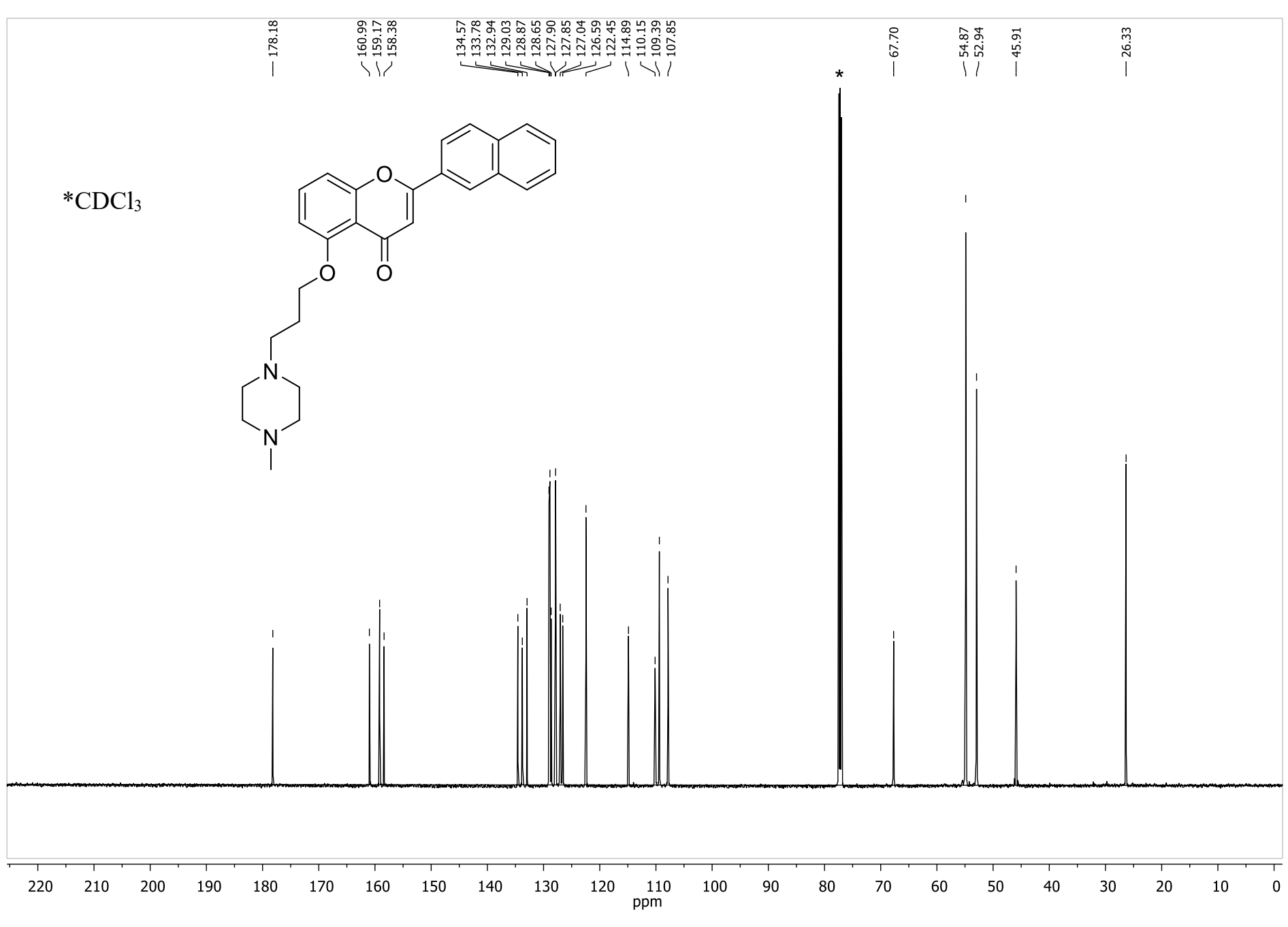

Supporting Information Figure S13. ^{13}C NMR of **3d**.

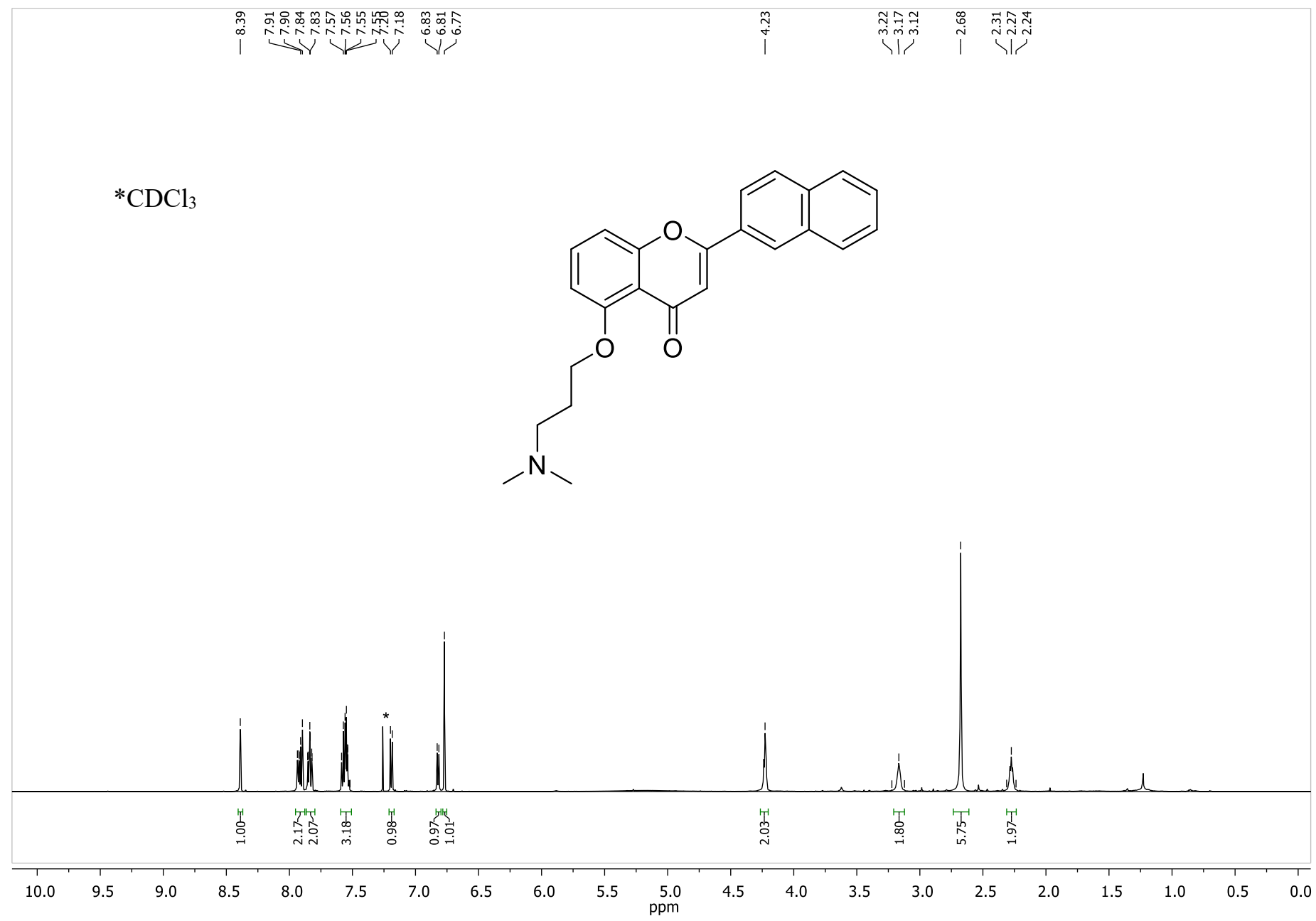

Supporting Information Figure S14. ^{1}H NMR of **3e**.

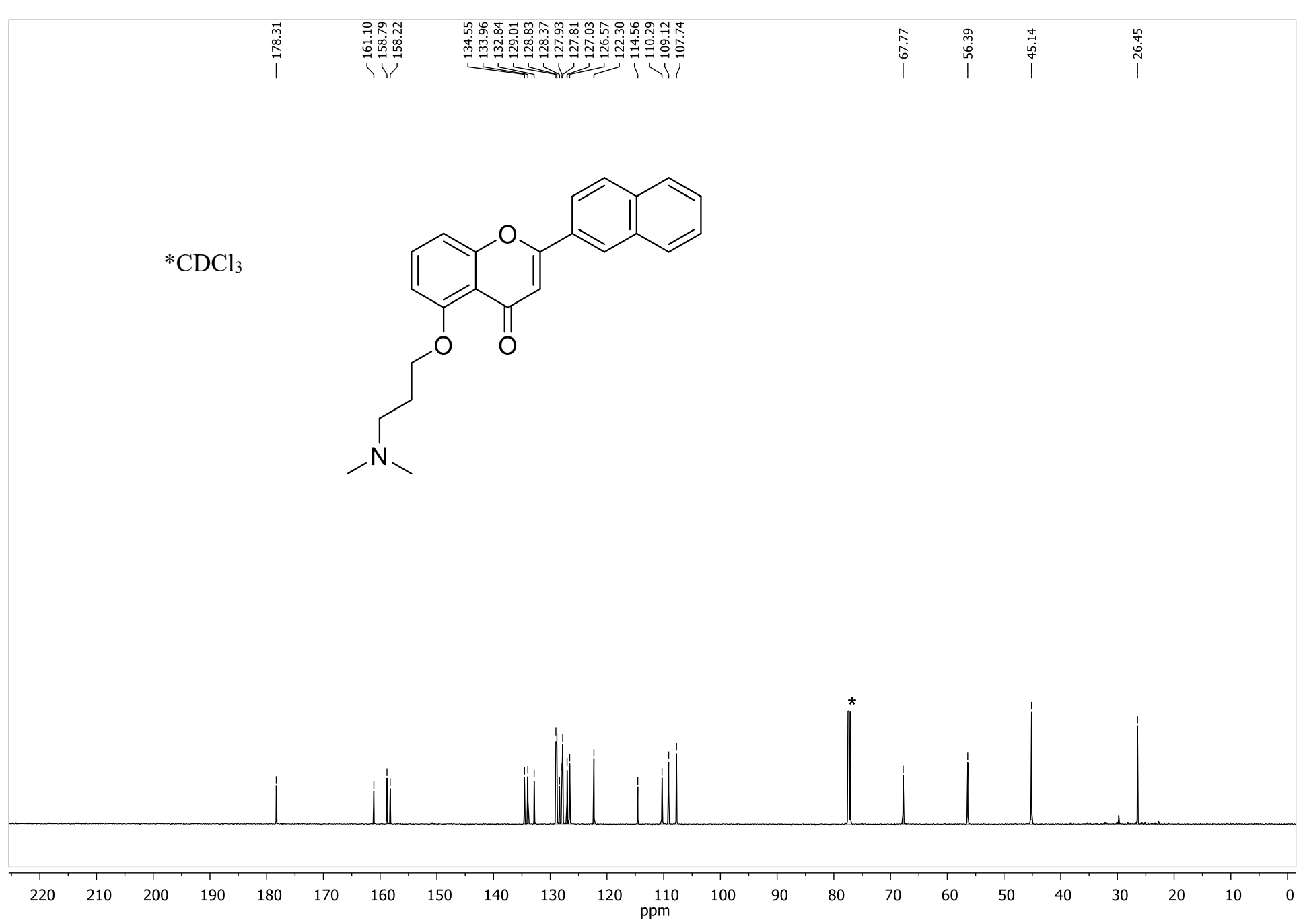

Supporting Information Figure S15. ^{13}C NMR of **3e**.

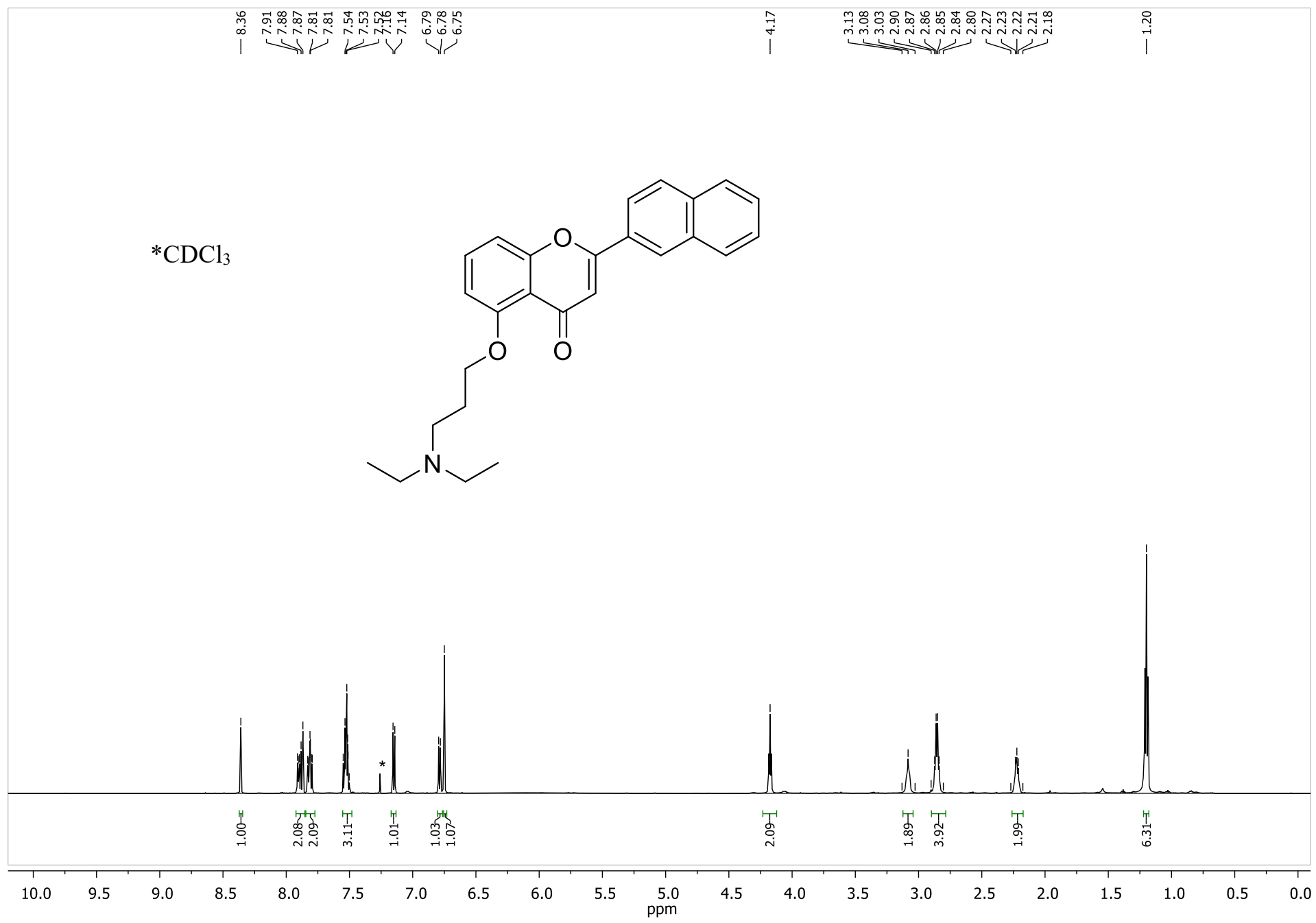

Supporting Information Figure S16. ^{1}H NMR of **3f**.

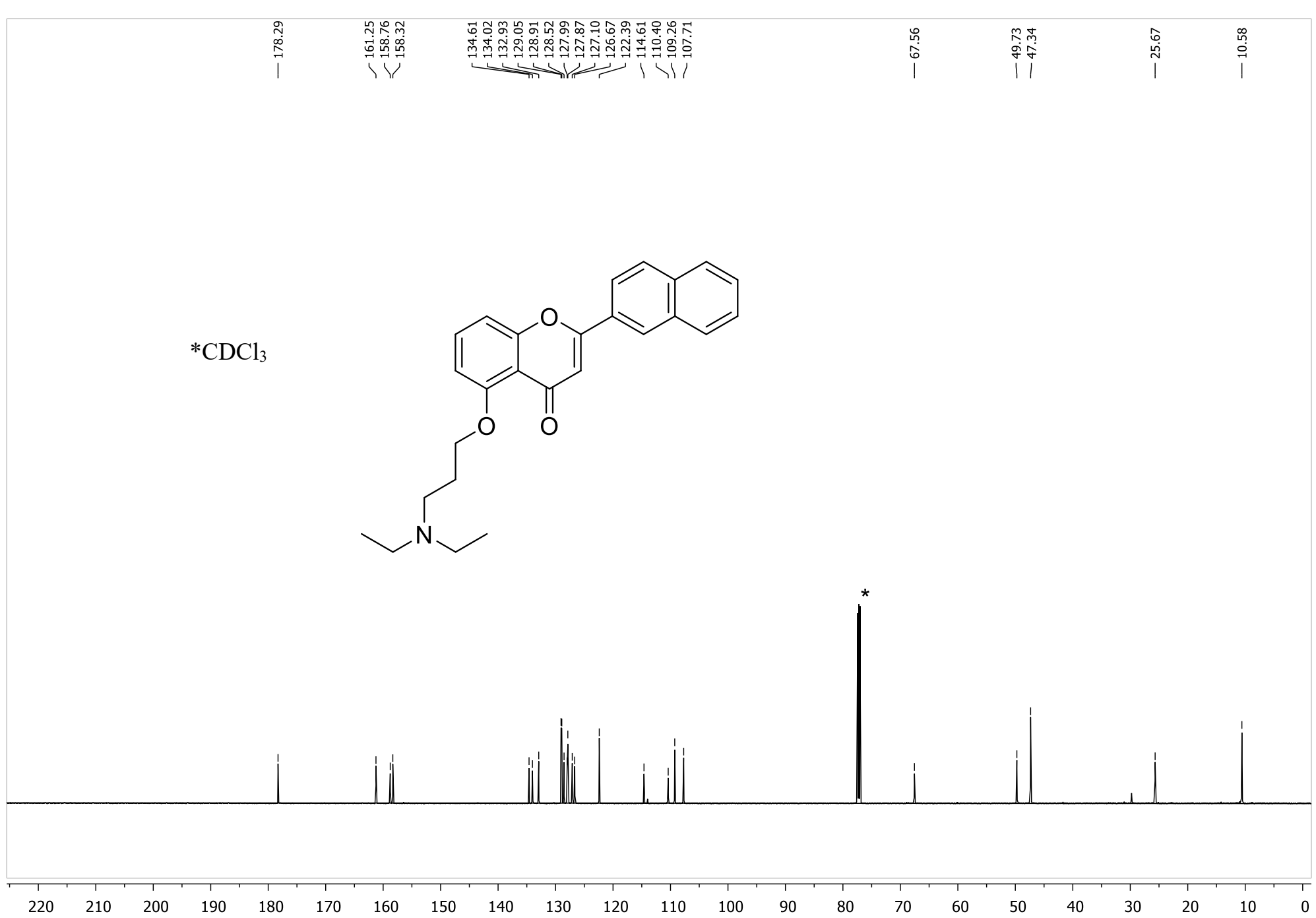

Supporting Information Figure S17. ^{13}C NMR of **3f**.

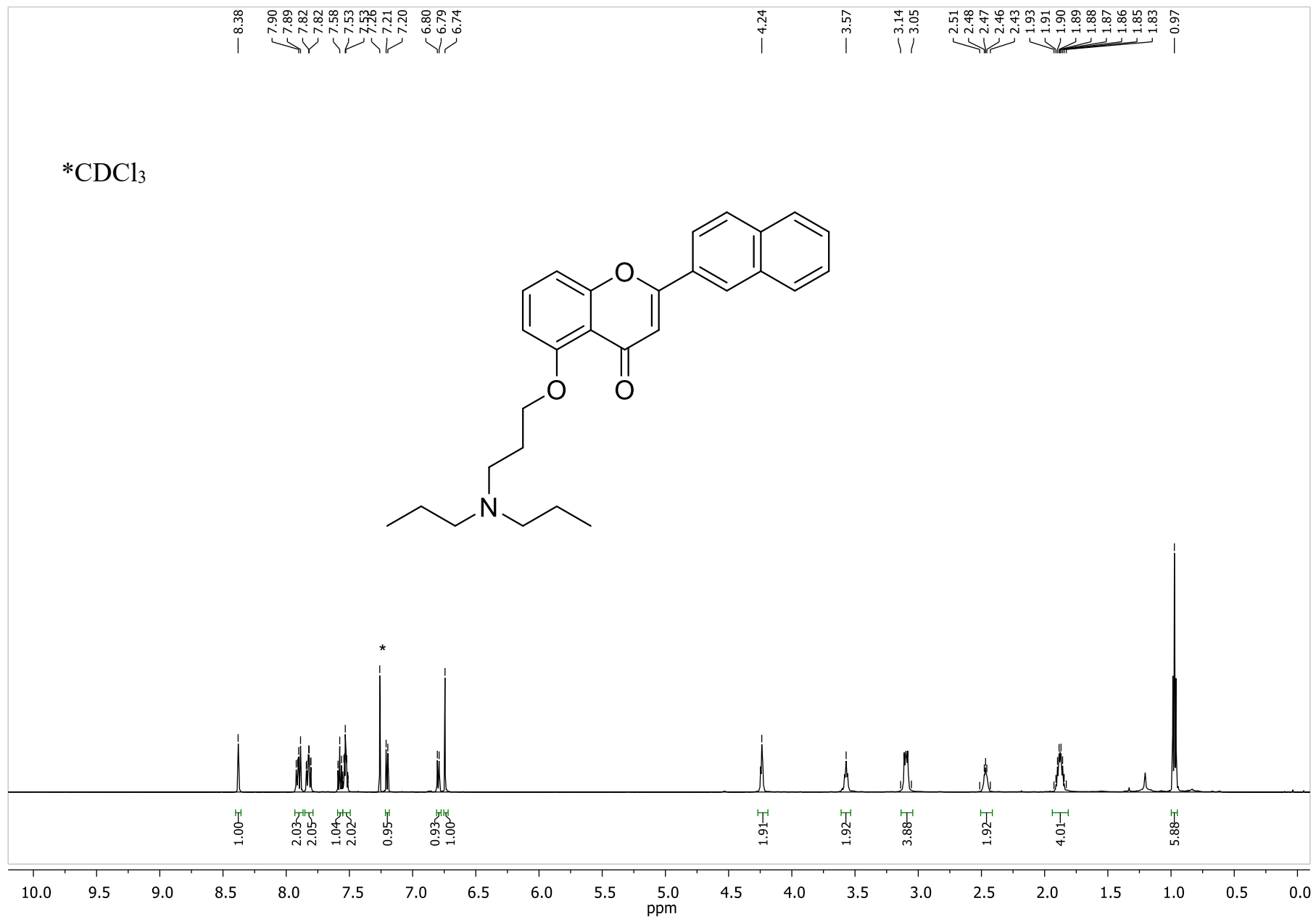

Supporting Information Figure S18. ^{1}H NMR of **3g**.

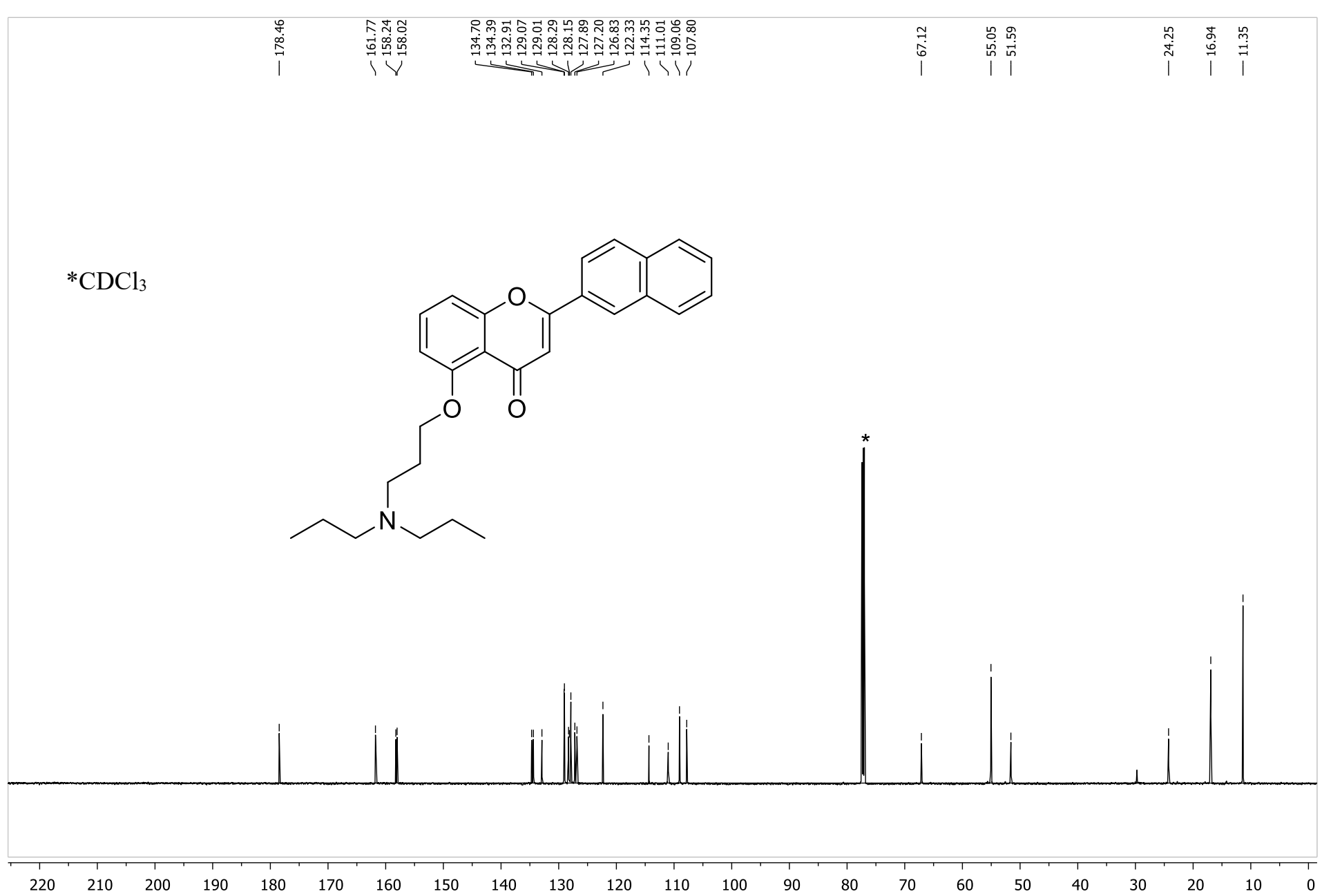

Supporting Information Figure S19. ^{13}C NMR of **3g**.

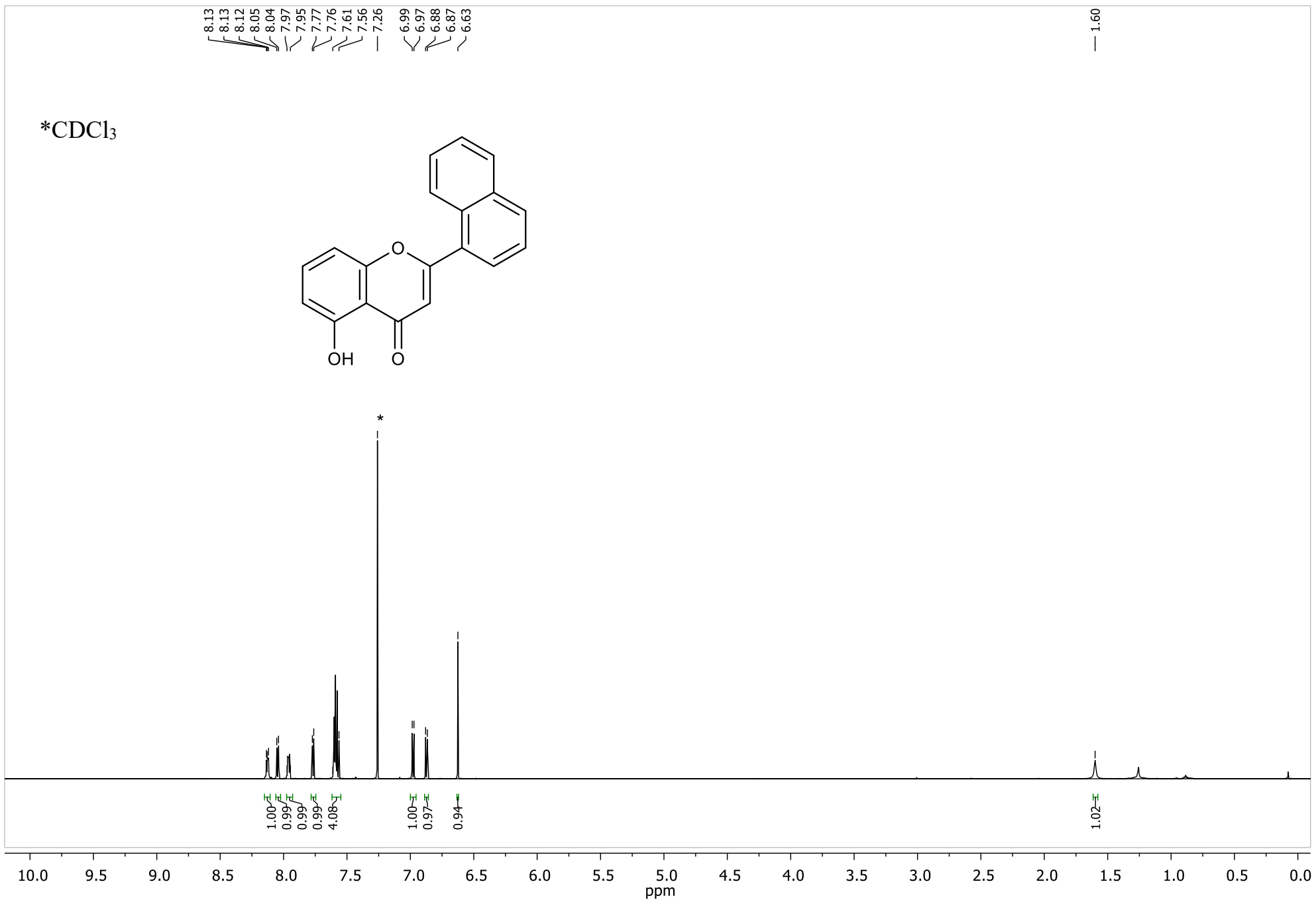

Supporting Information Figure S20. ^{1}H NMR of **6**.

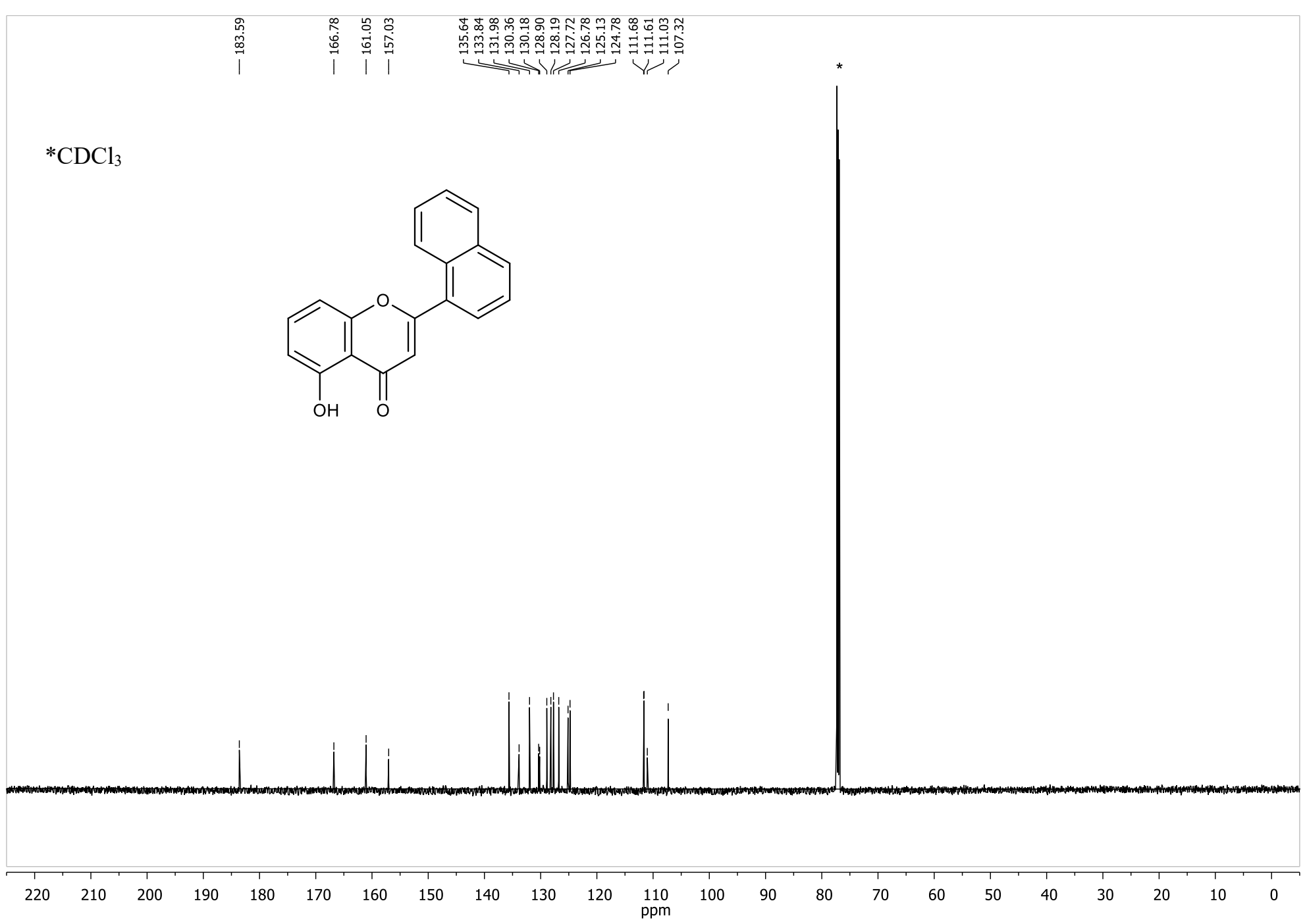

Supporting Information Figure S21. ^{13}C NMR of **6**.

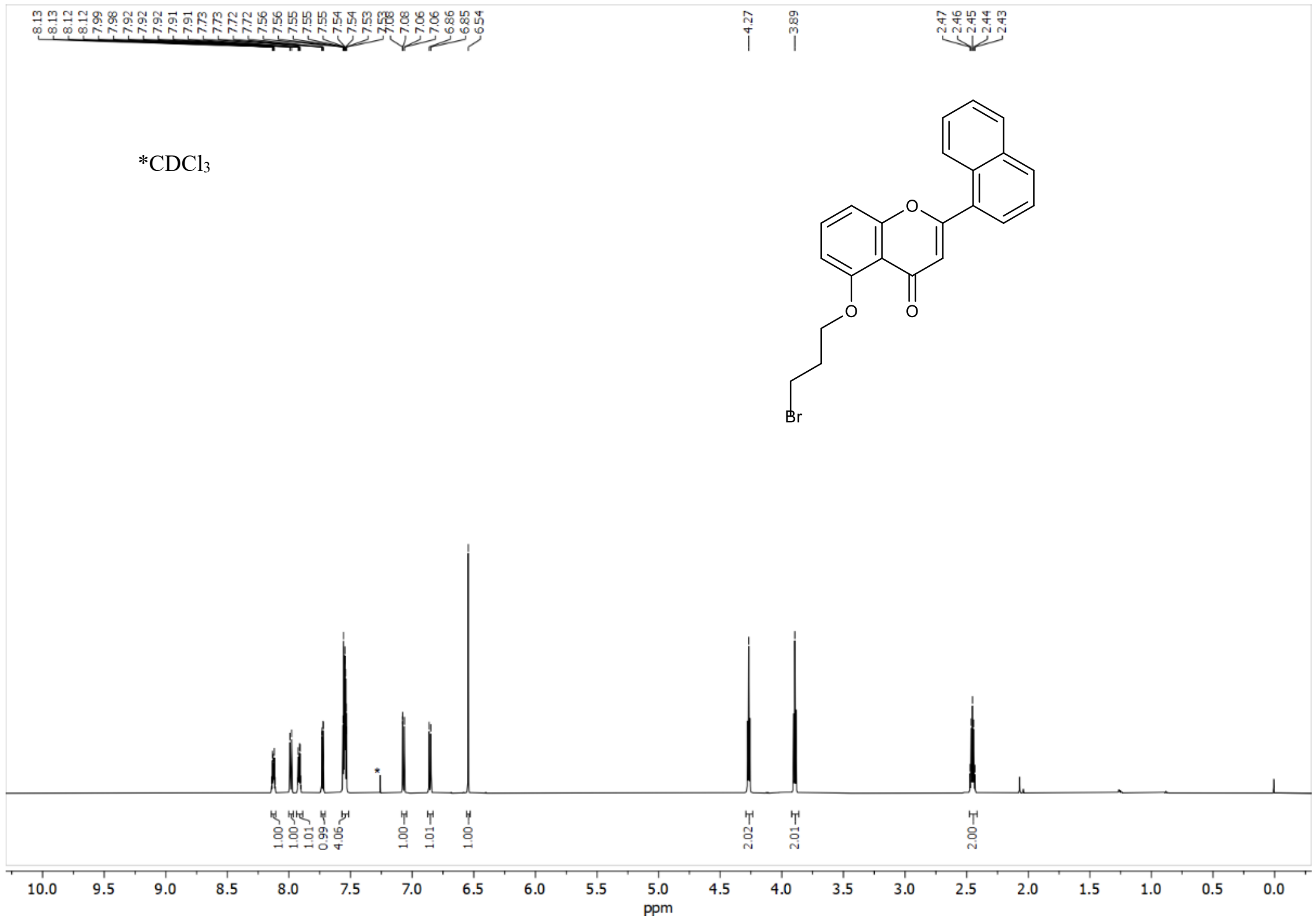

Supporting Information Figure S22. ^{1}H NMR of **7**.

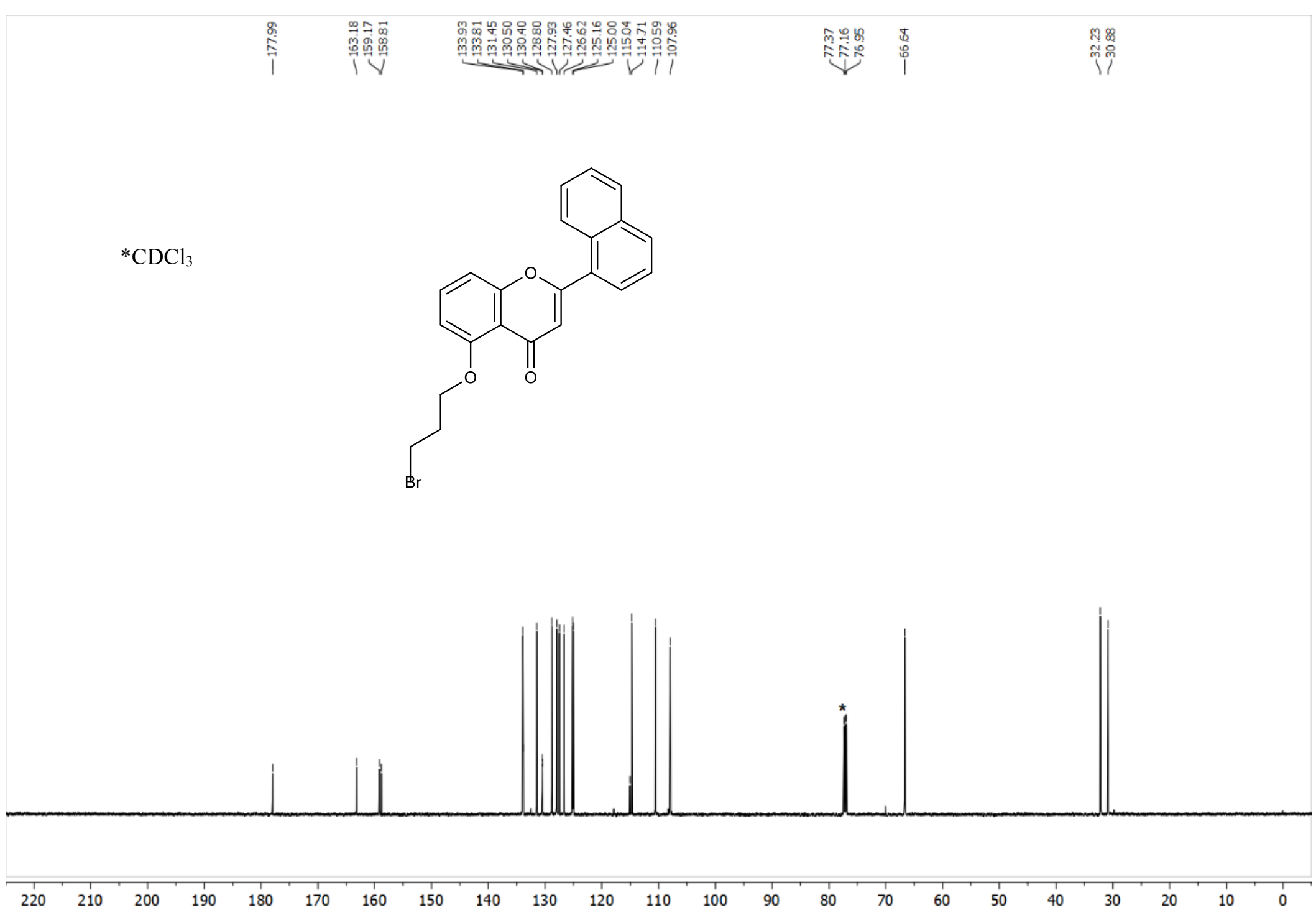

Supporting Information Figure S23. ^{13}C NMR of **7.**

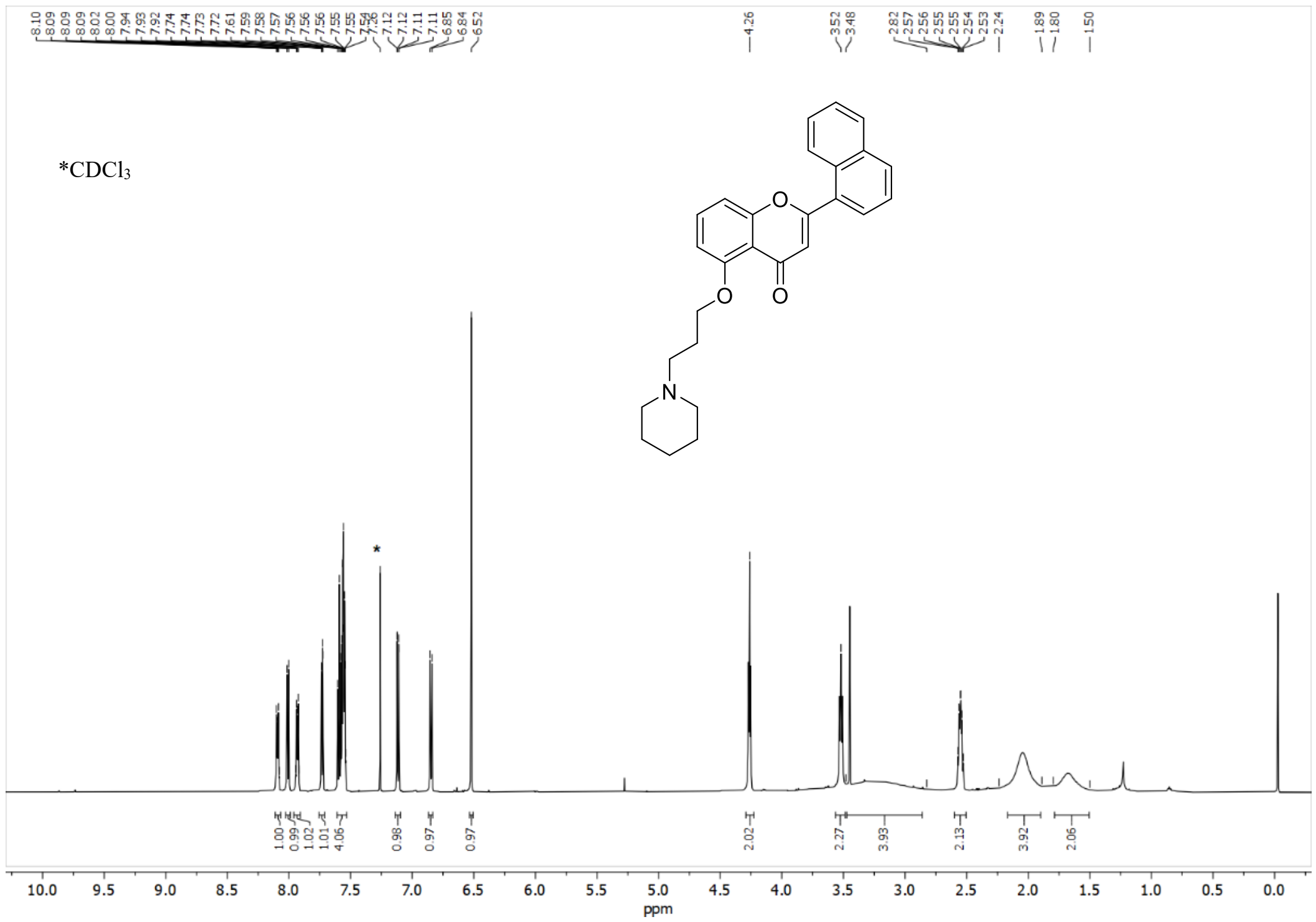

Supporting Information Figure S24. ^{1}H NMR of **4b**.

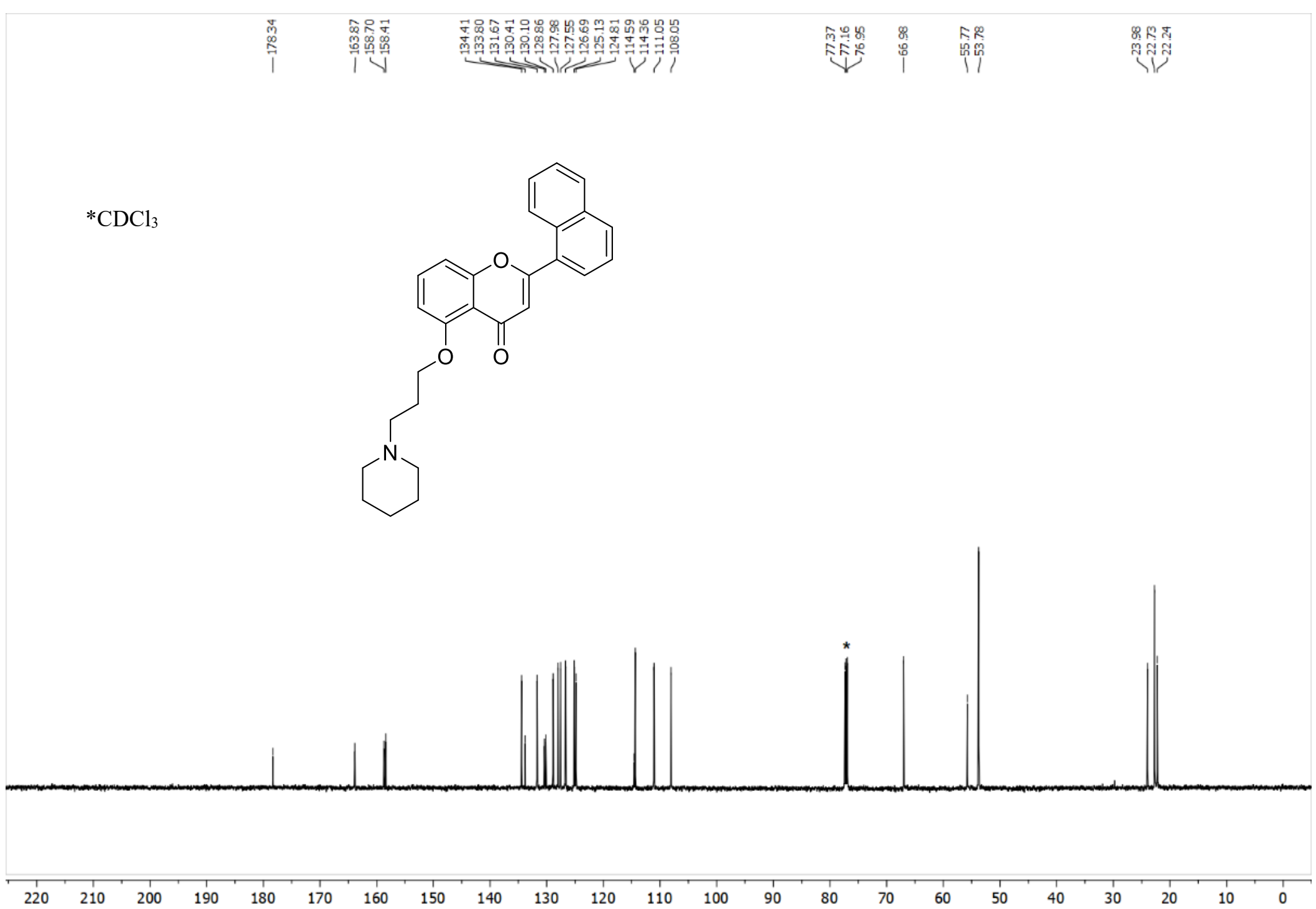

Supporting Information Figure S25. ^{13}C NMR of **4b**.
